# Extracellular matrix microarchitecture modulates cellular behavior and extracellular vesicle phenotypes in biomimetic tendon models

**DOI:** 10.1002/btm2.70134

**Published:** 2026-04-24

**Authors:** Kariman A. Shama, Zachary F. Greenberg, Evangeline M. Meyler, Emily M. Brown, Mei He, Brittany L. Taylor

**Affiliations:** ^1^ J. Crayton Pruitt Family Department of Biomedical Engineering University of Florida Gainesville Florida USA; ^2^ Department of Pharmaceutics University of Florida Gainesville Florida USA; ^3^ Department of Pharmacology and Therapeutics University of Florida Gainesville Florida USA

**Keywords:** extracellular matrix, extracellular vesicles, proteomics, tendinopathy, tendon

## Abstract

Tendon disease is a highly prevalent musculoskeletal disorder characterized by extracellular matrix (ECM) disorganization and fibroblast activation, contributing to fibrotic tissue healing and impaired function. While extracellular vesicles (EVs) have emerged as key mediators of intercellular communication and drivers of fibrosis in various tissues, their role in tendon pathology remains poorly understood. In this study, we developed a physiologically relevant 3D in vitro model that recapitulates biophysical features of healthy and fibrotic tendon microenvironments to investigate EV‐mediated contributions to tendon remodeling. Primary tendon‐derived cells on diseased scaffolds had increased proliferation, higher collagen III and fibronectin protein expression, and inferior cellular alignment. Proteomic profiling of EVs revealed temporally regulated, microenvironment‐dependent cargo reflective of disease progression in vitro. Diseased EVs were enriched in cytoskeletal, ECM‐remodeling, and inflammatory proteins, including vimentin (VIM), Annexin A2 (ANXA2), MMP2, and INHBA, suggesting an EV‐mediated role in promoting matrix remodeling, fibroblast activation, and chronic inflammation. Notably, the temporal analysis demonstrated the late‐stage emergence of stress‐responsive and myofibroblast‐associated proteins such as ENO1 and DES, underscoring the model's ability to capture the progressive nature of tendon pathology. In contrast, EVs from the healthy mimetic model demonstrated cargo associated with metabolic homeostasis and lipid transport, including APOA2 and CKM. Collectively, these findings highlight the utility of our tendon model as a dynamic platform for studying tendon pathology and establishing EVs as both sensitive indicators of microenvironmental state and potential mediators of fibrotic progression. This work provides a foundation for future studies exploring the diagnostic and therapeutic potential of EVs in tendinopathy.

## Graphical abstract

Using a biomaterials‐based 3D tendon model, this study shows that diseased‐mimetic matrices drive fibroblast activation and ECM remodeling. Extracellular vesicles (EVs) reflected disease stage, with EVs enriched in inflammatory and remodeling proteins, while healthy EVs carried homeostatic markers; highlighting their potential role as mediators and indicators of tendon pathology progression.

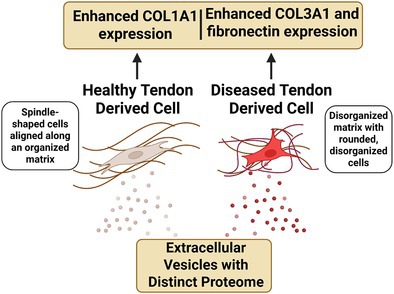

**Translational Impact Statement**

This work establishes advancements towards a representative 3D tendon model that mimics healthy and diseased microenvironments, enabling detailed study of tendinopathy progression. By revealing dynamic changes in tendon cell behavior and extracellular vesicle (EV) cargo linked to fibrosis and inflammation, our study identifies molecular signatures to inform more precise mechanistic therapeutic targets. Ultimately, this mechanistic understanding supports more targeted interventions to treat tendinopathy and tendon fibrosis, improving patient outcomes and restoring tendon function.

## 1 INTRODUCTION

Tendinopathy is a prevalent clinical condition characterized by pain, swelling, and impaired tendon function. The debilitating condition accounts for approximately 30% of musculoskeletal pain‐related visits in primary care settings,^1^, ^2^ and is commonly attributed to overuse injuries. Tendon‐related disorders manifest into chronic pain, reduced productivity, and diminished quality of life, resulting in a substantial socioeconomic burden.^3^ At the tissue level, tendinopathy is marked by structural, compositional, and cellular reprogramming changes. Healthy tendon exhibits highly organized collagen fibers sparsely populated by aligned tendon‐derived cells, whereas diseased tendon shows fragmented and disorganized collagen bundles, microvascular proliferation, neoinnervation, and alterations that ultimately reduce mechanical integrity.^4^, ^5^, ^6^, ^7^ Multiple studies demonstrate that tendinopathic tissues exhibit elevated collagen III expression and increased fibronectin levels relative to healthy controls.^8^, ^9^, ^10^ Additionally, collagen I upregulation during early tendon pathology has been reported in both rat and human models.^11^, ^12^ These tissue compositional changes impact cellular response. At the cellular level, tendon cells undergo changes during tendinopathy, as the tissue itself becomes hypercellular. The cells lose their spindle‐like structure and become rounded, ultimately impairing mechanotransduction and, therefore, tendon function.^13^ These pathological hallmarks underscore the importance of understanding tendon biology at the molecular level with the goal of developing more precise and effective therapeutics to address pathology rather than symptoms (i.e., administration of anti‐inflammatories, RICE method, orthotics).

Tendinopathy is a complex condition shaped by interactions between intrinsic and extrinsic cell populations. These populations contribute distinct cellular and molecular processes and communicate through signaling mechanisms that transfer functional molecules, such as proteins, modulating the behavior and phenotype of recipient cells. Extracellular vesicles (EVs) are small, membrane‐bound particles naturally released by cells that mediate such intercellular communication through the transfer of proteins, lipids, and genetic material.^14^ ^,^ ^15^ They are broadly categorized into exosomes, microvesicles, and apoptotic bodies, each originating from distinct cellular pathways and serving specific biological functions.^16^, ^17^ Small‐EVs contribute to both physiological and pathological processes, including immune modulation, tissue repair, and the progression of diseases such as cancers (e.g., breast, prostate, pancreatic) and fibrotic disorders (e.g., liver cirrhosis, pulmonary fibrosis).^14^, ^18^, ^19^, ^20^, ^21^ Emerging evidence suggests that EVs also contribute to musculoskeletal disorders by influencing inflammation, extracellular matrix remodeling, and cellular communication.^22^, ^23^, ^24^, ^25^ In the context of tendon biology, several studies have explored how EVs contribute to regenerative processes in the tendon, however, the precise mechanisms by which EVs contribute to tendon repair and the progression of tendinopathy remain poorly understood.^26^, ^27^, ^28^ Gaining deeper insight into their endogenous role within the native healing environment is critical for uncovering EV‐mediated mechanisms that lead to disease progression and phenotypes, thereby identifying novel molecular targets and developing precise, mechanism‐based interventions for tendon disorders. The capacity of EVs to carry bioactive cargo underlies their emerging potential as vehicles for targeted drug delivery and regenerative therapies.^29^

We previously showed that cellular proliferation and EV secretion increase in our in vitro tendon models, suggesting that scaffold mechanics and topographical cues may modulate EV and cellular function and contribute to tendon homeostasis.^30^ In the present study, we aim to deduce the temporal changes of EV cargo in response to homeostatic and diseased tendinous microenvironments while simultaneously validating the cellular phenotype to assess model fidelity. The objectives of this study were twofold: (1) to validate that tendon‐derived cells cultured on our biomimetic in vitro platforms exhibit a pathological matrix change consistent with tendinopathy, and (2) to leverage this model to conduct proteomic profiling of EVs for identifying signaling pathways associated with tendon pathology. We hypothesized that cells cultured on the diseased mimetic platform would demonstrate increased proliferation and elevated expression of tendon matrix remodeling markers, collagen I, III, and fibronectin, indicative of the tendinopathic phenotype. We also hypothesize that EVs secreted within the two microenvironments will have distinct phenotypes and proteomes.

To model tendon homeostasis and pathology, we previously developed complementary healthy and disease‐mimetic platforms that capture key structural and cellular features of tendinopathy.^30^ The healthy platform recapitulates aspects of native tendon architecture by incorporating highly aligned, thicker fibers that emulate normal tendon microstructure. In the present study, the healthy model supports spindle‐shaped nuclear morphology, organized extracellular matrix (ECM) deposition, and tendon‐like cellular elongation. In contrast, the disease‐mimetic platform was previously engineered with thinner, disorganized fibers that mimic the fibrotic topography of diseased tendons. We demonstrate here that these components of our platform drive hypercellularity, rounded cell morphology, aberrant alignment, and persistent upregulation of fibrotic ECM proteins such as fibronectin and collagen III. Both platforms support tendon‐derived cell growth and matrix production; however, their divergent fiber architecture and cellular responses provide a physiologically relevant basis for examining how tendon microenvironments regulate cell behavior and EV phenotypes. Unlike traditional models that utilize biochemical induction of pathology, our approach harnesses physical microenvironment, fiber orientation and stiffness, to emulate hallmark features of healthy and diseased tendons. Tendon‐like mechanical and structural cues modeled by our platform can significantly influence cellular behavior and their EV biogenesis. We are the first, to our knowledge, to report the development of a diseased platform that accurately captures the dynamic human signaling pathways that occur during tendinopathy. Our findings underscore the significance of our 3D in vitro healthy and diseased mimetic models as an advanced representative platform for investigating the interplay between microenvironmental cues, tendon cell behavior, and EV‐mediated contributions to tendon homeostasis and pathology.

## 2 METHODS AND MATERIALS

### 2.1 Scaffold fabrication

**FIGURE 1:**
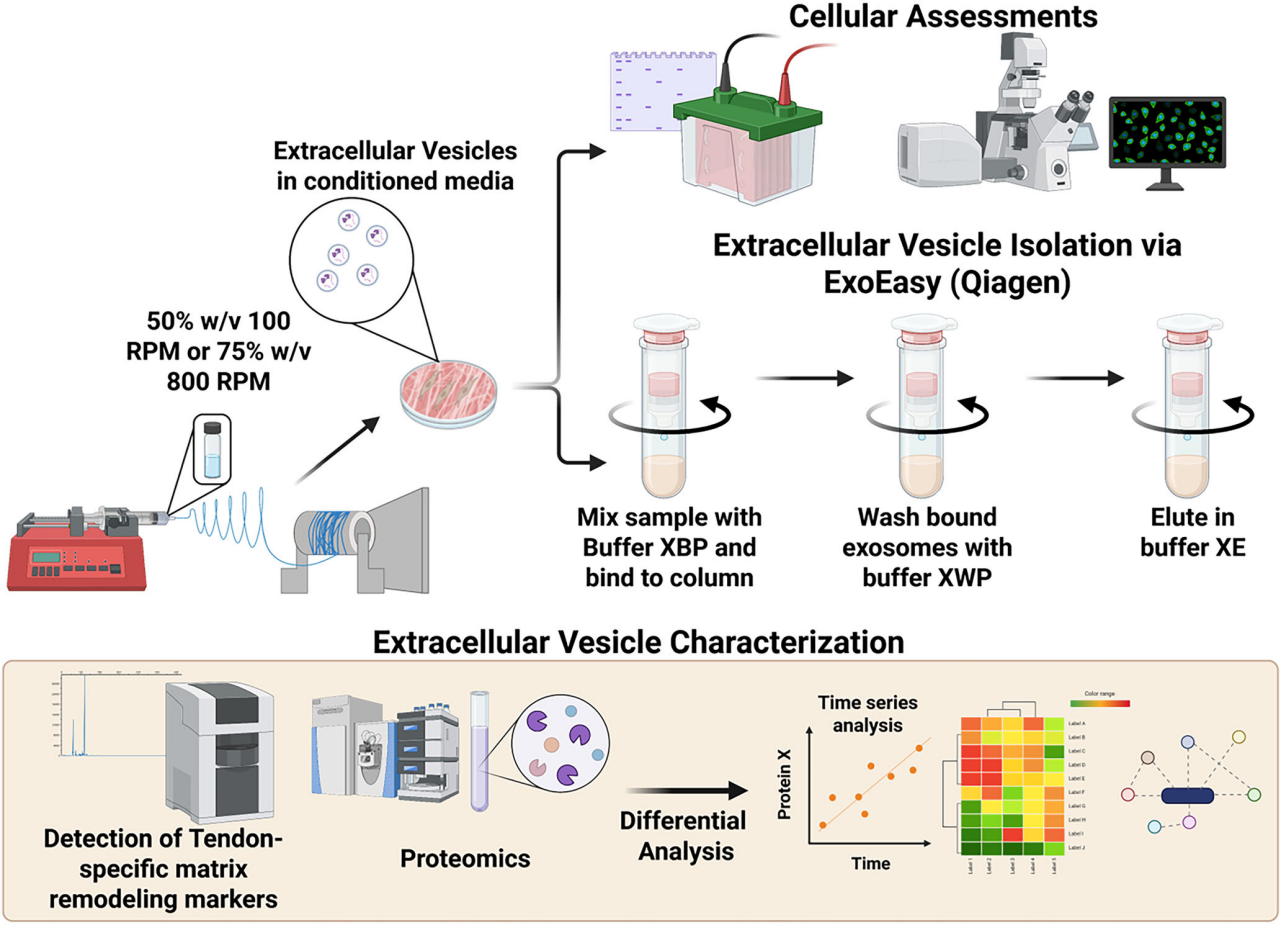
Schematic of extracellular vesicle (EV) sample preparation and experimental workflow for in vitro model validation. Polycaprolactone (PCL) solutions at 50% w/v and 75% w/v were electrospun onto a rotating mandrel at 100 and 800 RPM, respectively, to fabricate nanofibrous scaffolds. Primary tendon fibroblasts derived from Sprague–Dawley rats were seeded onto these scaffolds and cultured for 10 days. Western blotting and immunofluorescent staining were performed to assess the expression of tendon‐specific matrix remodeling proteins, as well as to evaluate cellular morphology and alignment. Conditioned media were collected for EV isolation using the ExoEasy kit (Qiagen). EV proteomic profiling was conducted. Expression of tendon‐specific remodeling markers within EVs was analyzed using a capillary electrophoresis‐based system.

The experimental workflow is depicted in Figure 1. Our in vitro platforms utilizing electrospun nanofibrous scaffolds were developed as previously described.^30^ Briefly, polycaprolactone (PCL) solutions (Thermo Scientific; Cat: 178305000; Fair Lawn, NJ, USA) were prepared by dissolving PCL in dichloromethane (Sigma‐Aldrich; Cat: 270997; St. Louis, MO, USA) and dimethylformamide (Thermo Scientific; Cat: 279600040; Fair Lawn, NJ, USA) overnight at room temperature to achieve 75% and 50% weight/volume (w/v) solutions. These solutions were subsequently electrospun onto a rotating cylindrical aluminum mandrel at speeds of either 100 or 800 revolutions per minute, positioned approximately 13 cm from the 16‐gauge needle tip. Electrospinning was performed under a high‐voltage power supply generating a voltage potential of 26 kV. The polymer extrusion rate was maintained at 5 mL/h, with ambient conditions set to a relative humidity range of 35%–46% and a temperature of 26°C.

### 2.2 Cell culture

All animal tissues used in this study were obtained from deceased male and female Sprague–Dawley rats provided by another research laboratory at our institution. No animals were euthanized specifically for the purpose of this study. All procedures involving the use of animal‐derived tissues were conducted in accordance with institutional guidelines for the ethical use of animal materials and complied with the policies of the Institutional Animal Care and Use Committee. Primary tendon‐derived cells were isolated from male and female 9‐month‐old Sprague–Dawley rats. We chose rats as the source of primary tendon cells because their tendon architecture, particularly fiber alignment, vascularity, and fascicular structure, more closely resembles that of human tendons compared to murine (mouse) models.^31^, ^32^ Despite interspecies differences, findings in rats have often translated well to human conditions, particularly when validating therapeutic targets in tendon.^33^, ^34^ The rat tendon‐derived cells were isolated from the Achilles tendon using an established cell isolation method that validates the cells are free of mycoplasma contamination. These cells are robust and represent the native cellular population responsible for tendon maintenance and remodeling. While our findings are based on a well‐established preclinical model, further validation using human tendon cells will be essential to ensure clinical relevance and translational potential. The primary tendon‐derived cells were cultured until passage 2 in low glucose Dulbecco's Modified Eagle Medium (Gibco; Cat. No. 11885084) supplemented with 20% exosome depleted fetal bovine serum (Gibco; Cat. No. A2720801) and 1% Penicillin–Streptomycin (Pen/Strep) under normoxic culture conditions (5% CO_2_, 37°C, pH 7 media, saturating humidity). Nanofibrous scaffolds were trimmed to match the diameter of a six‐well tissue culture plate, sterilized, and then seeded with passage 3 tendon‐derived cells at a density of 52,000 cells per square centimeter (*n* = 3–6). Media was collected on days 3, 5, 7, and 10 for EV isolation (*n* = 3–6). The isotropic modulus of our healthy model is 57.29 ± 7.671 MPa, whereas our diseased model has a modulus of 7.151 ± 1.066 MPa.^30^ Culture media was replenished at every timepoint to prevent contamination and the accumulation of waste products, ensuring a consistent and healthy environment for EV production. The timepoints were selected to capture crucial stages of tendinopathy: acute response (days 3–5), early matrix remodeling (day 7), and the consolidation of matrix remodeling (day 10).^35^, ^36^

### 2.3 Cell proliferation and immunofluorescent assays

Cellular quantification was performed using DNA staining, as previously described (*n* = 3) (Thermo Scientific™, Cat. No. 62249; Thermo Scientific, Waltham, MA, USA).^30^, ^37^ To evaluate cellular metabolic activity, an MTS assay was carried out (Promega, Cat. No. G3582; Madison, WI, USA) (*n* = 3) (Figure S4B). Cellular morphology was assessed via actin staining (Invitrogen, Cat. No. A12379) and nuclear counterstaining with DAPI (Fisher Scientific, Cat. No. H‐1200‐10) (*n* = 3). Nuclear aspect ratio was analyzed via ImageJ, version 1.54p. Briefly, images underwent thresholding to generate a binary mask over the DAPI stain. The nuclei of each cell underwent ellipse fitting using the “analyze particles” tool with the “fit ellipse” option selected. Aspect ratio is calculated by dividing the major axis, the longest diameter of the ellipse, by the diameter that is perpendicular, the minor axis. A minimum of 5–30 cells was quantified from at least three randomly selected fields per biological replicate, with *n* = 3 independent replicates per condition. Utilizing OrientationJ, an ImageJ plugin software, cellular orientation distribution was quantified by applying a structure tensor analysis to determine the dominant local orientation of image intensity gradients. The distribution of cells was then normalized to total pixel‐intensity distribution to determine the percent of total orientation signal between −90 and 90 degrees. Cellular alignment was determined utilizing an automated binarization‐based extraction of alignment score method as previously described.^38^ Briefly, images underwent preprocessing using median and band‐pass filters to remove noise, followed by local adaptive thresholding (Sauvola's algorithm) to binarize images and identify cells. Lastly, the extraction of cell orientation and area from objects was identified in binary images. The alignment score is computed by determining the weighted average cosine of twice the difference between individual cell orientations and a candidate overall alignment angle, thereby maximizing this average to find the dominant alignment direction and its strength score, which ranges from 0 to 1, effectively quantifying uniaxial alignment. An alignment score of 0 corresponds to randomly orientated cells, and 1 corresponds to perfect alignment.

### 2.4 Western blot

Protein samples were prepared by mixing in a 1:1 ratio with 5% β‐mercaptoethanol in 2× Laemmli buffer and subsequently denatured at 95°C for 10 min. Proteins were resolved by SDS‐PAGE using Any kD™ Mini‐PROTEAN® TGX™ Precast Protein Gels (Bio‐Rad; Cat. No. 4569033) at a constant voltage of 200 V for 35 min. Following electrophoresis, proteins were transferred onto PVDF membranes using a semi‐dry blotting system (Bio‐Rad; Cat. No. 1704156). Membranes were blocked at room temperature for 1 h using Intercept® Blocking Buffer (LI‐COR; Cat. No. 927‐70001) to minimize non‐specific binding. Following blocking, membranes were incubated overnight at 4°C with primary antibodies, each diluted 1:1000 in blocking buffer. The antibodies used included anti‐collagen I (Novus Biologicals; Cat. No. NBP1‐30054), anti‐collagen III (Cat. No. NB600‐594), anti‐fibronectin (Cat. No. NBP1‐91258), and anti‐beta‐actin (Cat. No. NB600‐501), all from Novus Biologicals. After washing, membranes were incubated with a Goat anti‐Rabbit IgG secondary antibody conjugated to IRDye® (LI‐COR; Cat. No. 926‐32211) at a 1:15,000 dilution for 1 h at room temperature (*n* = 6 per group). Signal detection was performed using an appropriate near‐infrared imaging system.

### 2.5 EV isolation and characterization

EVs were isolated from conditioned cell culture media using the ExoEasy™ kit (Qiagen; Cat. No. 76064) according to the manufacturer's protocol. Briefly, 5 mL of culture media was combined with an equal volume of XBP reagent, mixed, and centrifuged at 500 × g for 5 min at room temperature. The membrane was then washed with XWP reagent by centrifuging at 3000 × g for 5 min at room temperature, followed by the addition of 400 μL of XE reagent and a final centrifugation at 500 × g for 5 min at room temperature.

Isolated EVs underwent characterization following the guidelines set by the Minimal Information for the Studies of Extracellular Vesicles (Figure S8).^17^ Transmission electron microscopy was employed to evaluate EV morphology (Talos L120C, Thermo Scientific, Waltham, MA, USA) (Figure S8A). Nanoparticle tracking analysis (NTA) was performed using the Zetaview nanoparticle tracking analyzer (Particle Metrix, Ammersee, Germany) to determine the size distribution and concentration of EVs (*N* = 3 per group) (Figures S4A and S8B). EV samples were resuspended and diluted in cold PBS to achieve the optimal working concentration for the NTA system. Measurements were taken at 11 distinct positions using a 488 nm wavelength for each sample. Tendon remodeling markers collagen I, collagen III and fibronectin expression were analyzed (*N* = 3 per group) using an automated capillary electrophoresis system (JESS, ProteinSimple, San Jose, CA, USA) (Figure S8C). Band intensity was measured using ImageJ.

### 2.6 Proteomics

Tendon‐derived EVs proteins were extracted and digested using the EasyPep™ MS Sample Prep Kit (Thermo Fisher Scientific). Total protein was determined on a Qubit and the appropriate volume of each sample was taken to equal 20 μg total protein for digestion. The samples were digested with sequencing grade trypsin/lys C rapid digestion kit from Promega (Madison WI) using manufacturer recommended protocol. Three times the sample volume of rapid digestion buffer (provided with the kit) was added to the samples. The sample was incubated at 56°C with 1 μL of dithiothreitol solution (0.1 M in 100 mM ammonium bicarbonate) for 30 min prior to the addition of 0.54 μL of 55 mM Iodoacetamide in 100 mM ammonium bicarbonate. Iodoacetamide was incubated at room temperature in the dark for 30 min. The trypsin/lys C was prepared fresh as 1 μg/μL in the rapid digestion buffer. One microliter of enzyme was added and the samples were incubated at 70°C for 1 h. The digestion was stopped with the addition of 0.5% TFA. The MS analysis is immediately performed to ensure high quality tryptic peptides with minimal non‐specific cleavage.

Nano‐liquid chromatography tandem mass spectrometry (Nano‐LC/MS/MS) was performed on a Thermo Scientific Q Exactive HF Orbitrap mass spectrometer equipped with an EASY Spray nanospray source (Thermo Scientific) operated in positive ion mode. The LC system was an UltiMate™ 3000 RSLCnano system from Thermo Scientific. The mobile phase A was water containing 0.1% formic acid and the mobile phase B was acetonitrile with 0.1% formic acid. The mobile phase A for the loading pump was water containing 0.1% trifluoracetic acid. Five milliliter of sample is injected on to a Thermo Sientific mPAC^ä^ C18 trapping column (C18, 5 μm pillar diameter, 10 mm length, 2.5 μm inter‐pillar distance). at 10 μL/mL flow rate. This was held for 3 min and washed with 1%B to desalt and concentrate the peptides. The injector port was switched to inject and the peptides were eluted off of the trap onto the column. Thermo Scientific 110 cm mPAC^ä^ was used for chromatographic separations (C18, 5 μm pillar diameter, 110 cm length, 2.5 μm inter‐pillar distance). The column temperature was maintained 40°C. A flowrate of 750 nL/min was used for the first 15 min and then the flow was reduced to 250 nL/min. Peptides were eluted directly off the column into the Q Exactive system using a gradient of 1% B to 20%B over 100 min and then to 45%B in 20 min for a total run time of 150 min: The MS/MS was acquired according to standard conditions established in the lab. The EASY Spray source operated with a spray voltage of 1.5 kV and a capillary temperature of 200°C. The scan sequence of the mass spectrometer was based on the original TopTen™ method; the analysis was programmed for a full scan recorded between 375 and 1575 Da at 60,000 resolution, and a MS/MS scan at resolution 15,000 to generate product ion spectra to determine amino acid sequence in consecutive instrument scans of the 15 most abundant peaks in the spectrum. The AGC Target ion number was set at 3e6 ions for full scan and 2e5 ions for MS^2^ mode. Maximum ion injection time was set at 50 ms for full scan and 55 ms for MS^2^ mode. Micro scan number was set at 1 for both full scan and MS^2^ scan. The HCD fragmentation energy (N)CE/stepped NCE was set to 28 and an isolation window of 4 m/z. Singly charged ions were excluded from MS^2^. Dynamic exclusion was enabled with a repeat count of 1 within 15 s and to exclude isotopes. A Siloxane background peak at 445.12003 was used as the internal lock mass.

All MS/MS spectra were analyzed using the Peaks 12.5 (Bioinformatic Solutions, Guelph, ON, Canada). Rattus novegicus (sp_incl_isoforms TaxID = 10116_and_subtaxonomies) (v2024‐10‐02) and Universal Protein Contaminants fasta^39^ assuming the digestion enzyme trypsin. Peaks were searched with a fragment ion mass tolerance of 0.020 Da and a precursor ion tolerance of 10.0 ppm. Carbamidomethyl of cysteine was specified as a fixed modification and oxidation of Methione as a variable modification. Spectra obtained from all EV proteomics libraries were aggregated and batch corrected by ComBat.^40^

Next, all Rattus Novegicus proteins were mapped to their ortholog Homo Sapien protein through the ExoMEGA dictionary, followed by adjusting for sparsity by removing proteins with <25% zeros for values across the samples. Differential analysis testing was performed through the DEqMS library.^41^, ^42^ DEqMS leverages peptide count‐based empirical Bayes with Limma to estimate the fixed prior under a negative binomial distribution, calculating a moderated *t*‐statistic to determine differential proteins across groups. The data input into DEqMS is log2(protein intensities +1), along with their associated peptide counts for the between‐group comparison. gProfiler2,^43^ Cytoscape,^44^ EnrichmentMap,^45^ and clustermaker2^46^ were used to perform gene ontology analysis and pathway clustering with the GLay algorithm^47^ after time‐ series analysis to output the tendon derived cells functional landscape.

### 2.7 Statistics

Statistical analyses were conducted using GraphPad Prism 10 software and R v4.5. Group comparisons were made at each time point, and temporal changes in EV secretion were quantitatively measured. Two‐way analysis of variance (ANOVA) with Tukey's post hoc tests and normality assessments was applied to datasets where a temporal aspect was a factor. Protein expression data analysis at each time point underwent a one‐way ANOVA with Tukey's post‐hoc test. Nuclear aspect ratio and alignment score data underwent nonparametric statistical analysis using the Kruskal–Wallis test with Dunn's post‐hoc testing. For proteomic analysis, differentially expressed proteins were identified using a false discovery rate threshold of <0.05.^48^ The analysis of covariance was performed to evaluate the protein abundance rate between healthy and diseased tendon. Significance was defined as *p* < 0.05 (*), *p* < 0.01 (**), *p* < 0.001 (***), *p* < 0.0001 (****).

## 3 RESULTS

### 3.1 Healthy and diseased tendon‐mimetic models differentially influence tendon‐derived cell shape, growth, and alignment

**FIGURE 2:**
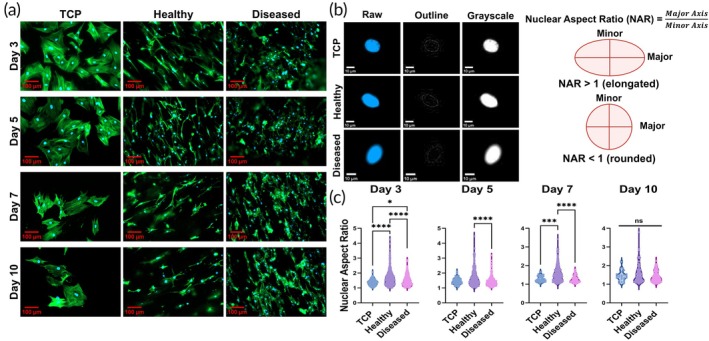
(a) Actin (green) and DAPI (blue) stain of tendon derived cells seeded on tissue culture plate (TCP) monolayer controls, healthy mimetic models and diseased mimetic models (*n* = 3). Scale bar: 100 μm. (b) Nuclear aspect ratio (NAR) was quantified using ImageJ by converting microscopic image to grayscale and measuring major and minor axes of individual nuclei. A higher major‐to‐minor ratio indicates increased cellular elongations. (c) Cells on the healthy model showed greater elongation than both the monolayer control and diseased model by day 3, while the diseased model also exceeded the monolayer control. By day 5, elongation remained elevated only in the healthy model relative to the diseased, and by day 7, the healthy group exhibited significantly greater elongation than all other models. No significant differences were observed by day 10. Significance was defined as *p* < 0.05 (*), *p* < 0.01 (**), *p* < 0.001 (***), *p* < 0.0001 (****), ns = not significant.

**FIGURE 3:**
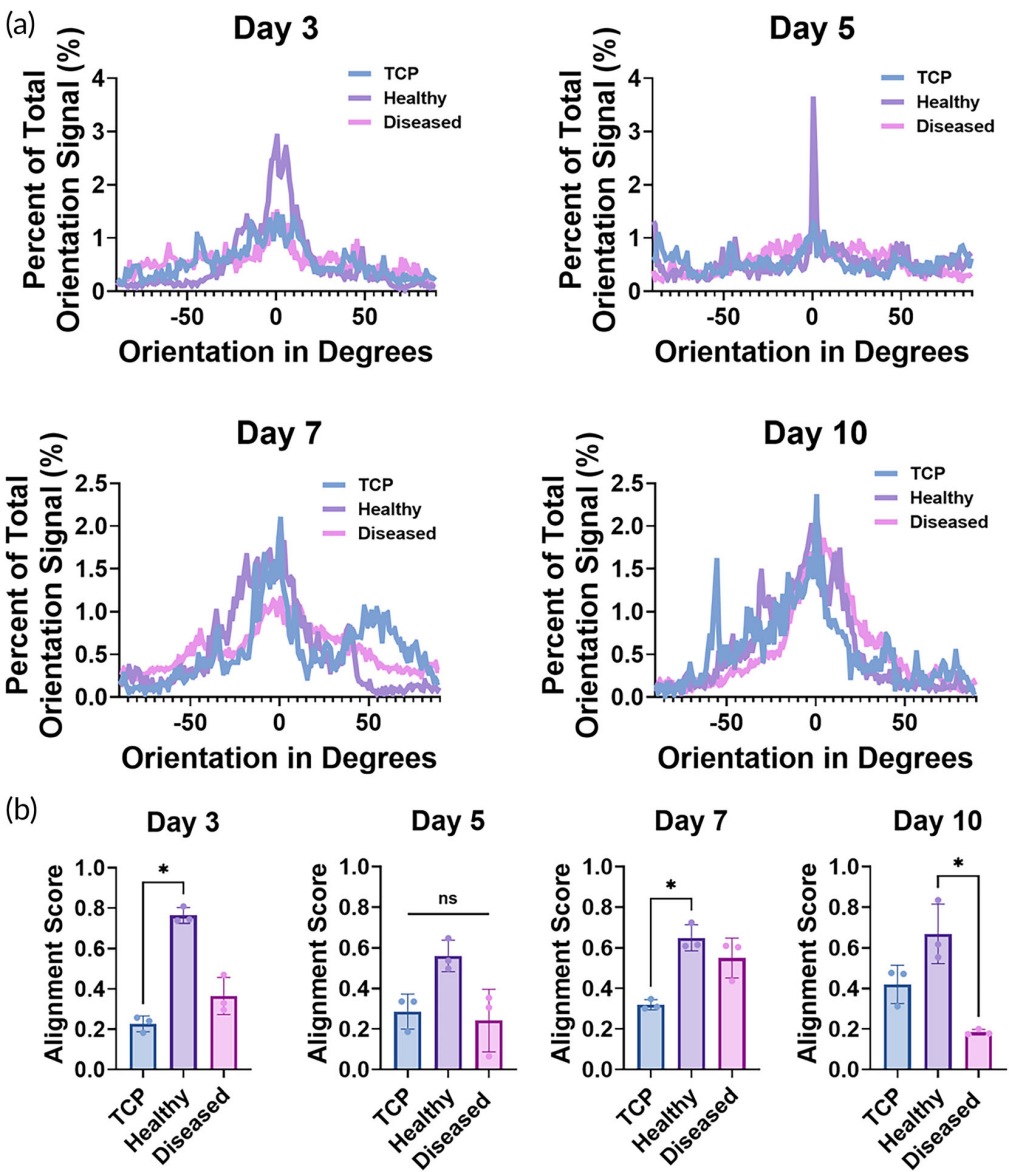
(a) Cellular orientation within each model was assessed using the OrientationJ plugin in ImageJ, with orientation angles ranging from −90° to 90°. (b) Alignment score was determined utilizing a binarization‐based extraction of alignment score (BEAS) method, where a score of 0 corresponds to randomly oriented cells and a score of 1 indicates highly aligned cells. Significance was defined as *p* < 0.05 (*), *p* < 0.01 (**), *p* < 0.001 (***), *p* < 0.0001 (****), ns = not significant. Data are expressed as mean ± standard deviation.

Previously, we employed an immortalized murine fibroblast cell line to model tendon pathology and observed enhanced cell proliferation in diseased mimetic conditions relative to monolayer controls. Building on these findings, we now utilize a more physiologically relevant cell source, primary tendon‐derived cells. Cells were seeded onto tissue culture plate monolayers, as well as healthy and diseased mimetic scaffolds, and assessed via nuclear and actin staining (Figure 2a). Qualitative observations revealed increased cellular elongation on healthy mimetic scaffolds. To quantify this, we measured nuclear aspect ratio (NAR), defined as the ratio of the major to minor nuclear axis, using ImageJ. Tendon‐derived cells on healthy mimetic scaffolds exhibited significantly greater NAR values compared to both monolayer and diseased conditions as early as day 3, a trend that continued on day 7. By day 5, cells on healthy mimetics showed increased elongation relative to only our diseased models (Figure 2b,c). Additionally, nuclear staining demonstrated enhanced cell proliferation in diseased mimetic models compared to healthy models by day 10, aligning with the elevated cellular proliferation observed in tendinopathy (Figure S4C). We utilized the OrientationJ plugin in ImageJ to quantitatively assess cellular orientation as a percent of total orientation signal ranging from angles −90° to +90° (Figure 3a). A pronounced peak centered around 0°, representing the neutral axis, indicates strong directional alignment. The sharpness and height of this peak reflect the degree of alignment, with taller and narrower peaks signifying a higher proportion of cells oriented consistently along a common axis. By day 3, cells within the healthy scaffold models exhibited significantly greater alignment compared to both the monolayer control, a trend that was also reflected on day 7 (Figure 3b). The healthy models demonstrated markedly enhanced cellular alignment relative to the diseased models at day 10. Interestingly, on day 5, we saw no significant variations in alignment score.

### 3.2 Temporal induction of tendon ECM proteins in disease‐mimetic models

**FIGURE 4:**
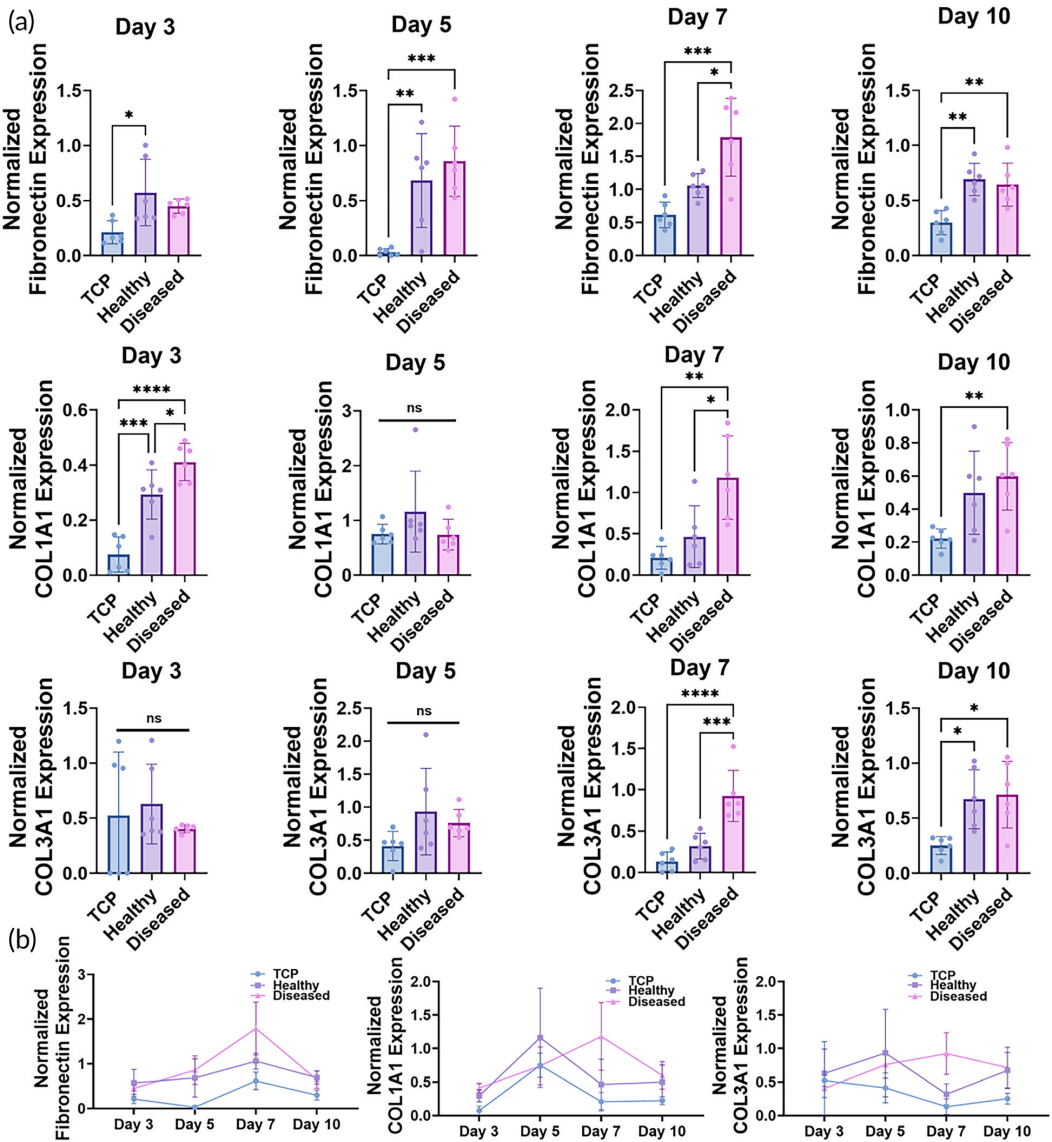
(a) Quantified graphs of fibronectin, collagen I, and collagen III expression, normalized to β‐Actin (*n* = 6). (b) Protein expression of fibronectin, collagen I, and collagen III over 10 days in culture. Significance was defined as *p* < 0.05 (*), *p* < 0.01 (**), *p* < 0.001 (***), *p* < 0.0001 (****), ns = not significant. Data are expressed as mean ± standard deviation. Western blot membranes showing the expression levels of fibronectin, collagen I, collagen III, and β‐actin in tendon fibroblasts isolated from our models at each timepoint are presented in Data S1.

Western blot analysis revealed significant temporal changes in the expression of tendon matrix remodeling markers, collagen I, fibronectin, and collagen III (Figures S1, 2A–C). Fibronectin expression in diseased mimetic models increased in a time‐dependent manner, with a marked rise between days 3 and 5, and peak levels observed at day 7 (Figures S2A, Figure 4b). By day 7, fibronectin expression in the diseased group was significantly higher than both the monolayer control and healthy mimetic models, consistent with patterns observed in tendinopathy (Figure 4a). Collagen I expression also varied over time across all groups, peaking at day 5 in healthy mimetic models and at day 7 in diseased mimetic models (Figures 4a,b and S2B). For collagen III, the diseased mimetic group exhibited a progressive increase in expression, reaching a maximum at day 7 (Figures 4a and S2C). Notably, collagen III levels were significantly elevated in the diseased group compared to both controls at days 7 and 10.

### 3.3 EVs reflect microenvironment‐dependent expression of tendon matrix remodeling proteins

**FIGURE 5:**
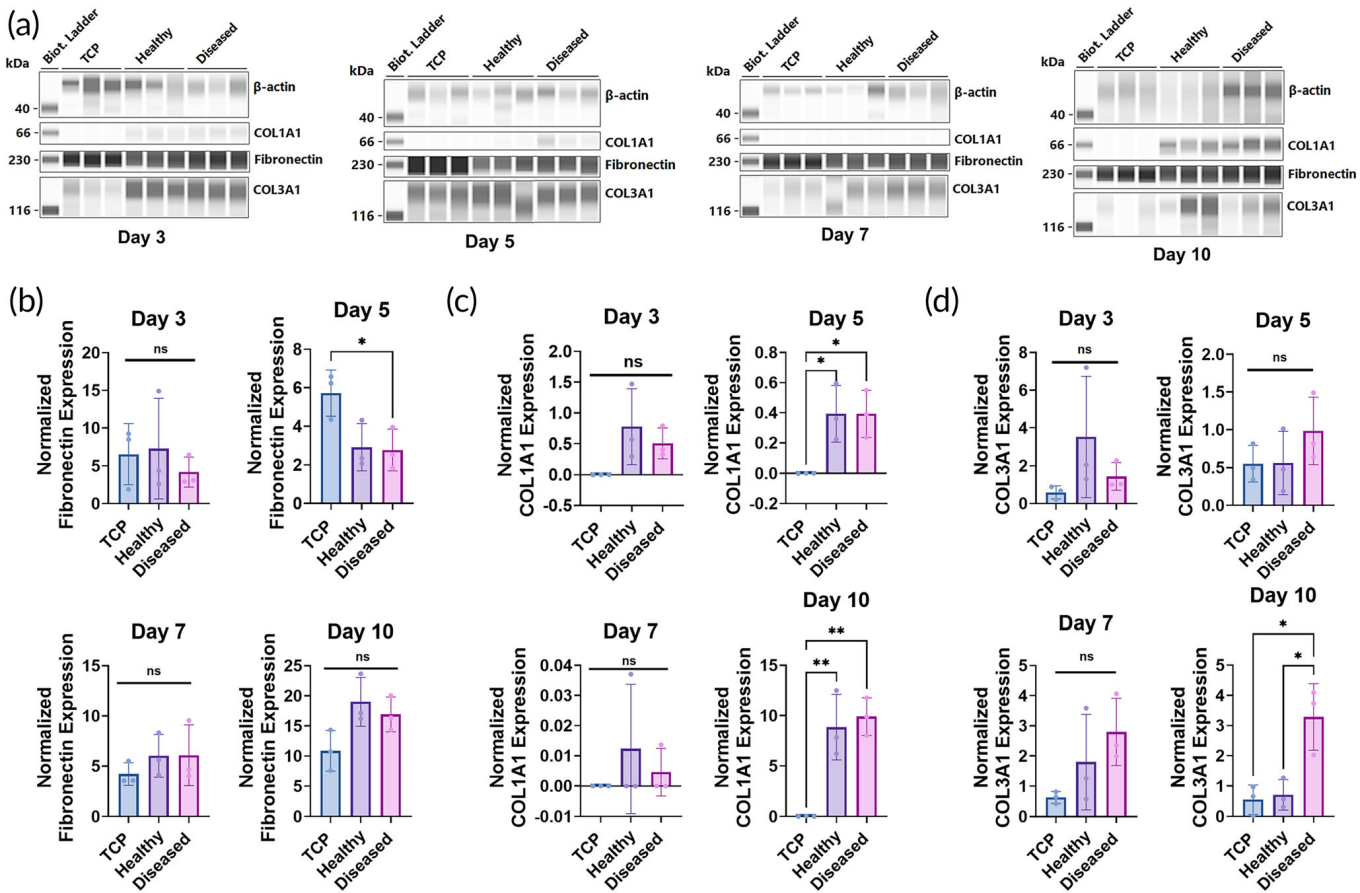
(a) Representative capillary electrophoresis profiles showing fibronectin, collagen I, collagen III, and β‐actin in extracellular vesicles (EVs) isolated from each model condition. Quantification of (b) fibronectin, (c) collagen I, and (d) collagen III expression levels, each normalized to β‐actin. *p* < 0.05 (*), *p* < 0.01 (**), *p* < 0.001 (***), *p* < 0.0001 (****), ns = not significant. Data are expressed as mean ± standard deviation.

Protein expression of tendon‐specific matrix remodeling markers in EVs was analyzed using a capillary electrophoresis‐based system (Figures 5a and S5). Notably, fibronectin expression was significantly higher in the monolayer control compared to the diseased model by day 5, suggesting reduced fibronectin association with EVs under fibrotic conditions (Figure 5b). In contrast, collagen I expression was significantly elevated in both the healthy and diseased tendon‐mimetic models compared to the monolayer control at days 5 and 10, with no significant difference observed between the two models (Figure 5c). Collagen III expression exhibited a distinct pattern, with significantly greater levels detected in EVs from the diseased model relative to all other conditions by day 10, indicating enhanced matrix remodeling activity associated with fibrotic microenvironments (Figure 5d). We observed a significant, time‐dependent increase in fibronectin levels in both healthy and diseased EVs, whereas collagen I and collagen III did not display significant temporal changes in expression (Figure S3).

### 3.4 EV proteomic signatures reflect dynamic inflammatory and matrix remodeling responses to diseased tendon microenvironments

**FIGURE 6:**
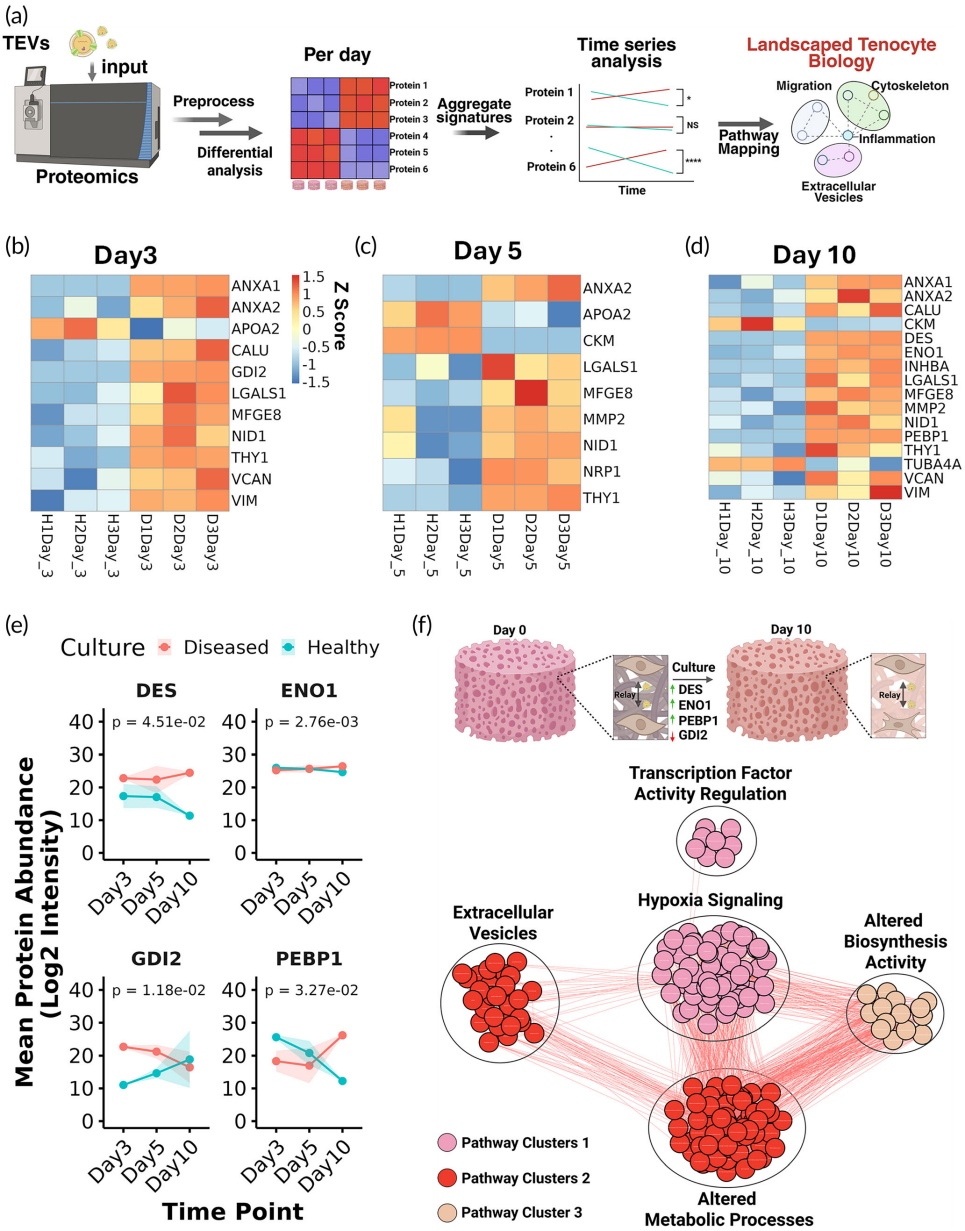
(a) Schematic representation of the proteomic analysis workflow. (b–d) Heatmaps depicting differentially enriched proteins at day 3, day 5, and day 10 respectively. (e) Time‐course analysis with an ANCOVA to identify the most statistically significant proteins that changed over time, highlighting their temporal regulation. (f) EnrichmentMap visualization of enriched biological process clusters mediated by tendon‐derived EVs, illustrating functional relationships among temporally regulated proteins.

We performed proteomic profiling to identify differentially expressed proteins in EVs derived from healthy and diseased microenvironments, followed by time‐series analysis and pathway mapping to characterize the functional landscape of tendon‐derived cells (Figure 6a). The heatmap shows temporal protein expression in EVs secreted from tendon‐derived cells cultured on healthy and diseased mimic scaffolds for 10 days. On day 3, EVs in the diseased model were upregulated in cytoskeletal proteins (VIM, ANXA2), ECM‐associated proteins (THY1, VCAN, NID1), and immunomodulators (ANXA1, LGALS1, MFGE8) (Figure 6b). EVs from the healthy model were observed to be enriched in APOA2 (days 3 and 5). On day 5, diseased EVs also had persistent high levels of expression of ECM remodeling proteins (NID1, ANXA2) and immunomodulatory factors (LGALS1, MFGE8, MMP2) (Figure 6c). The heatmap showing enriched proteins at day 7 is included in the supplemental materials (Figure S7). On day 10, diseased EVs were enriched for proteins involved in inflammation (ANXA1, LGALS1, MFGE8, PEBP1, MMP2), cytoskeletal dynamics (ANXA2, DES, VIM), ECM adhesion (VCAN, NID1), and stress/metabolism (ENO1). Interestingly, growth, inflammation, and fibrosis‐associated INHBA was significantly enriched in day 10 diseased EVs. Although PEBP1, DES, and ENO1 expression was not significantly different between the healthy group compared to the diseased group at days 3 and 5, this trend was fully reversed by day 10, along with expression of CKM and TUBA4A (Figures 6d, e and S6). Despite GDI2 being significantly elevated on day 3, we showed that the disease group significantly reduced GDI2's protein abundance. We visualized DES, ENO1, GDI2, and PEBP1's functional pathways (Table S1) to show that these proteins in tendinopathy affect EV regulation, hypoxia signaling, altered transcriptional factor regulation, and biosynthesis activity (Figure 6f).

## 4 DISCUSSION

Our findings demonstrate that tendon‐derived cells exhibit distinct morphological, proliferative, and alignment behaviors in response to engineered microenvironments that mimic healthy and diseased tendon conditions.^49^, ^50^ Building on our previous work using immortalized murine fibroblasts, the use of primary rat tendon‐derived cells in this study provides a more physiologically relevant model, yielding insights that better reflect the in vivo cellular response to tendon injury and repair. Tendon‐derived cells cultured on healthy mimetic scaffolds displayed increased elongation along with their nuclei relative to both the monolayer control and the diseased mimetic model, as quantified by nuclear aspect ratio (NAR), consistent with the spindle‐shaped morphology typically observed in native tendon tissues.^51^, ^52^ This enhanced elongation occurred as early as day 3 over all groups and persisted through day 7 relative to the monolayer control and diseased group, indicating that scaffold architecture and mechanical cues in the healthy condition effectively promote tendon‐derived cell‐like morphology. In contrast, cells on diseased mimetic scaffolds exhibited lower NAR values relative to the healthy mimetic model, consistent with a more rounded, activated fibroblast phenotype commonly associated with fibrotic or inflamed tendon tissue.^53^, ^54^, ^55^ By day 10, no significant variations in NAR were observed among groups, albeit a subset of cells in the healthy model appeared highly spindle. We attribute this occurrence to the overall mean likely converging across groups due to cellular adaptation and matrix remodeling over time, a phenomenon observed previously in cell‐matrix interactions in synthetic fibrous hydrogels.^56^ The enhanced cell proliferation observed in the diseased mimetic model by day 10 further supports the fibrotic nature of this microenvironment. This is consistent with clinical and experimental findings where tendon injury is associated with hypercellularity and increased fibroblast proliferation, which contribute to excessive matrix deposition and tissue stiffening.^13^, ^57^ These changes likely reflect the pathological progression of tendinopathy, characterized by increased cellular density and altered cell‐matrix interactions. Analysis of cellular alignment revealed dynamic changes in response to scaffold cues. Cells on healthy mimetic scaffolds exhibited strong alignment along the scaffold fiber axis as early as day 3, a trend that persisted on day 7 and became more pronounced by day 10 when compared to the diseased models, aligning with the organized collagen fiber structure of native homeostatic tendon. Interestingly, there were no significant variations on day 5, suggesting a transient plateau in cytoskeletal organization before alignment diverged at later time points. This biphasic alignment pattern suggests that cells within the microenvironment undergo gradual reorganization, potentially influenced by changes in scaffold stiffness, matrix composition, or autocrine signaling over time.^58^, ^59^ These results collectively underscore the importance of microenvironmental context in modulating tendon‐derived cell behavior. Healthy scaffolds foster a morphology and alignment pattern consistent with homeostatic tendon tissue, while diseased scaffolds drive enhanced proliferation and reduced fiber alignment indicative of fibrotic remodeling. These distinct cellular responses highlight the utility of our model for studying tendon disease mechanisms and testing therapeutic strategies aimed at restoring cellular and matrix organization.

The observed temporal changes in protein expression of tendon ECM remodeling markers provide further support for the utility of our 3D diseased mimetic model in recapitulating hallmark features of tendinopathy. Specifically, fibronectin expression in the diseased condition demonstrated a clear time‐dependent upregulation, with peak levels at day 7, significantly exceeding those in both the healthy and monolayer controls. This pattern aligns with previous studies reporting elevated fibronectin levels in injured or degenerative tendon tissues, where fibronectin accumulation contributes to aberrant matrix architecture, altered mechanotransduction, and increased fibroblast activation.^60^, ^61^ In addition, the increased collagen I expression observed in both the healthy and diseased mimetic conditions by day 3 suggests an early remodeling response characteristic of tendon repair.^62^ Collagen I is the predominant fibrillar collagen in healthy tendon, and its upregulation may reflect an attempt to restore matrix integrity following mechanical or inflammatory challenge.^63^, ^64^ However, the sustained and significantly elevated collagen III expression in the diseased group, especially at days 7 and 10, provides a distinguishing feature of pathological remodeling. Collagen III is typically expressed at low levels in healthy tendon but is markedly upregulated in fibrotic or degenerative tendon, where it contributes to disorganized matrix deposition and impaired biomechanical function.^8^, ^9^, ^65^ Together, these data suggest that the diseased mimetic model induces temporally regulated ECM protein expression patterns reflective of progressive tendon pathology. The delayed yet persistent upregulation of fibronectin and collagen III may serve as molecular signatures of chronic fibrotic remodeling, driven by altered microenvironmental cues such as scaffold architecture, cytokine signaling, and matrix stiffness. These results not only underscore the physiological relevance of our model but also highlight its utility for probing tendon disease mechanisms and identifying potential therapeutic targets aimed at modulating ECM turnover and restoring tissue homeostasis.

Our analysis of tendon matrix remodeling proteins in EVs revealed differential protein cargo profiles depending on the microenvironmental conditions, highlighting the potential of EVs as sensitive reporters of tissue remodeling status. The reduced fibronectin expression observed in EVs derived from the diseased model compared to the monolayer control suggests a downregulation or altered trafficking of this glycoprotein under fibrotic conditions. Fibronectin is essential for ECM assembly, cell adhesion, and migration, and its dysregulation has been implicated in various fibrotic diseases, including tendon fibrosis.^66^, ^67^ The diminished presence of fibronectin in EVs may reflect disrupted matrix organization or aberrant EV packaging mechanisms associated with the fibrotic tendon microenvironment. In contrast, the upregulation of collagen I in EVs from both healthy and diseased tendon‐mimetic cultures compared to monolayer controls may imply that three‐dimensional (3D) culture conditions more closely mimic the native tendon environment, prompting cells to secrete EVs enriched in this primary structural component.^68^, ^69^ The lack of significant difference in collagen I levels between healthy and diseased conditions suggests that its EV‐associated expression is more dependent on matrix dimensionality and cellular mechanotransduction rather than fibrotic signaling alone. Collagen III, however, showed a more disease‐specific enrichment. By day 10, EVs from the diseased model contained significantly higher levels of collagen III compared to all other groups. This finding aligns with previous reports indicating that collagen III is upregulated during pathological remodeling in fibrotic tendon, contributing to scar‐like ECM deposition and impaired mechanical properties.^70^, ^71^ The elevated export of collagen III via EVs may reflect cellular efforts to modulate ECM composition or dispose of excess matrix proteins in response to a dysregulated microenvironment.^72^, ^73^ Taken together, these data support the concept that EVs serve as mirrors of dynamic ECM remodeling and may be leveraged as non‐invasive biomarkers for detecting fibrotic progression or evaluating the performance of tissue‐engineered tendon constructs. Future studies should explore the molecular mechanisms governing selective protein sorting into EVs under fibrotic stress, as well as the functional implications of these cargo differences in cell–cell communication and matrix remodeling.^74^, ^75^ The proteomic profiling of EVs derived from tendon cell cultures grown on healthy and diseased mimetic scaffolds reveals temporally dynamic differences in protein cargo that reflect distinct cellular responses to the microenvironment. These differences suggest that EVs serve as sensitive reporters of matrix state and may play an active role in perpetuating or resolving pathological tendon remodeling.

This study provides a comprehensive temporal proteomics analysis of EVs secreted by tendon‐derived cells cultured on biomimetic models of healthy and diseased tendinous microenvironments. The dynamic shifts in EV cargo over the course of 10 days reveal distinct molecular signatures associated with matrix remodeling, cytoskeletal reorganization, immune modulation and stress responses, all being processes that are pivotal to tendon homeostasis and fibrosis. EVs derived from the diseased mimetic model exhibited an early and sustained enrichment of structural and ECM‐associated proteins such as VIM, ANXA2, THY1, VCAN and NID1, particularly apparent on days 3 and 5. These findings align with prior work showing activated tendon fibroblasts in fibrotic microenvironment upregulate cytoskeletal remodeling and ECM deposition markers.^76^, ^77^ Vimentin (VIM) and Annexin A2 (ANXA2) are markers of cytoskeletal remodeling and are upregulated during epithelial‐to‐mesenchymal (EMT) in cancer tumoral progression and tissue injury responses.^78^, ^79^ Their presence in EVs may be indicative of not only cytoskeletal reorganization but also increased cellular plasticity of tendon‐derived cells in diseased microenvironments. In the context of tendon fibrosis, vimentin is a marker for fibroblast activation, which occurs especially in the proliferative stages of tendon pathology. Vimentin knockout studies also suggest that vimentin filaments bind and stabilize collagen type I mRNAs, with decreased collagen I expression in vimentin knockout fibroblasts.^80^ The elevated levels of ECM‐related proteins versican (VCAN) and nidogen‐1 (NID1) in diseased EVs reflect early ECM remodeling and matrix deposition, hallmarks of tendinopathy and scar formation. Increased versican content has been linked to tendinopathy, while nidogen‐1 serves as a basement membrane component within tendon tissue.^81^, ^82^

The diseased EVs also had elevated expression of immunomodulatory proteins Annexin A1 (ANXA1), MMP2 and galectin‐1 (LGALS1) which are key influencers of cellular signaling, resolution of inflammation and fibrosis.^83^, ^84^, ^85^ MMP2 exhibits collagenolytic activity, primarily by degrading smaller collagen fragments and cleaving denatured collagens as well as type IV collagen.^86^ In a rabbit Achilles tendon overuse model, MMP2 activity in serum was significantly increased after 1 week of repetitive motion, suggesting MMP2 is activated during early stages of tendinopathy.^87^ The observed enrichment of MMP2 within the diseased EVs at both day 5 and day 10 underscores the pathological relevance of our diseased mimetic model, and highlights EV potential role in tendon degeneration by promoting temporal matrix remodeling and collagen degradation. Interestingly, we also saw enhanced enrichment of MFGE8, a protein enriched in exosomes to mediate uptake by recipient cells.^88^ The sustained expression of these proteins across all timepoints could be indicative of the immunosuppressive effects on the diseased tendinous microenvironment, potentially promoting fibrotic remodeling. An interesting finding was the elevated expression of INHBA, a subunit of activin A implicated in fibroblast activation, TGF‐β signaling, and tissue fibrosis, in EVs from the diseased models by day 10.^89^ The emergence of INHBA as a significant EV cargo during the late stage of the study further cements the notion of progressive fibrotic remodeling and may be indicative of an EV‐based marker of chronic tendinopathy. Furthermore, the concurrent upregulation of VIM and ANXA1 in the diseased EVs by day 10, both associated with wound response and myofibroblast activation, supports the notion that our diseased mimetic models drive an early pro‐fibrotic and inflammatory phenotype in tendon cells, which is reflected in their EV cargo.^90^, ^91^, ^92^, ^93^

By day 10, heatmaps indicated the diseased EV proteome displayed conspicuous markers of stress‐responsive and metabolic proteins such as enolase‐1 (ENO1). ENO1 is a glycolytic enzyme that is upregulated during cellular stress and has been previously implicated in fibrotic diseases and tumor progression.^94^, ^95^ On the contrary, EVs from the healthy mimetic model were enriched in muscle creatine kinase (CKM) and apolipoprotein A‐II (APOA2) at early timepoints. CKM is a metabolic enzyme that is associated with energy homeostasis, whereas APOA2 has been identified in EVs previously and plays a role in lipid transport and cholesterol homeostasis.^96^, ^97^, ^98^ The enrichment of ENO1 in the diseased EVs by day 10 highlights the metabolic reprogramming characteristic of the pathologic tendon microenvironment, whereas the expression of CKM and APOA2 early in the healthy mimetic derived EVs could be emblematic of EV populations contributing to the maintenance of metabolic balance within the tendon microenvironment.

Time‐course analysis revealed the most statistically significant proteins that changed over time in EVs isolated from the healthy and diseased models. Significant differences in desmin (DES), ENO1, GDP Dissociation Inhibitor 2 (GDI2), and Phosphatidylethanolamine‐binding protein 1 (PEBP1) expression between healthy and diseased groups were identified. DES is variably expressed in myofibroblasts, which are elevated during tendinopathy.^99^, ^100^ Additionally, DES is associated with regions of neovascularization and neoinnervation, features that are more pronounced in tendinopathy and tendon fibrosis.^101^, ^102^, ^103^ As a component of the intermediate filament network in muscle cells, DES contributes to mechanotransduction^104^ and in the context of tendon biology may serve as a biomarker of cellular differentiation as tendon cells transition toward a myofibroblast‐like phenotype.^105^ By day 10, DES was significantly enriched in diseased EVs, suggesting EV‐mediated signaling may reflect or contribute to this pathological shift. We also saw a significant elevation of glycolytic enzyme ENO1 over time in the diseased EVs. ENO1 drives metabolic reprogramming in fibroblasts in fibrosis and is also implicated in hypoxia.^106^, ^107^ Over time, it was elevated in the diseased group, most likely attributed to increased energy demands associated with ECM synthesis in tendinopathy. Interestingly, there was significant enrichment of GDI2 (GDP Dissociation Inhibitor 2) in the diseased EVs at day 3 and 5, but this trend flipped by day 10. GDI2 is a protein that modulates the activity of Rab GTPases, crucial regulators of intracellular vesicle trafficking and has been identified as a diagnostic biomarker for hepatocellular carcinoma.^108^ This observed early enrichment in the diseased EVs may reflect a dynamic regulation of vesicle trafficking processes during progression of tendinopathy. PEBP1 is a known regulator of the Raf/MEK/ERK and NF‐κB signaling pathways, pathways implicated in inflammation and fibrotic responses.^96^, ^109^ Associated with amplifying mitochondrial stress and contributing to tissue dysfunction and disease progression in pathological contexts, PEBP1 showed a significant increase over time in the diseased group.^110^ Similar to ENO1, we postulate that this increase is most likely reflective of the heightened energy demands necessary to support the changing diseased tendinous microenvironment.

Our in vitro platform reflects how EV protein cargo dynamically changes as the 3D tendon disease models mature from day 0 to day 10, highlighting temporal regulation of cell–cell signaling relevant to disease modeling. Pathway clustering following time‐course analysis demonstrates the tendon cell functional landscape, which includes EVs, altered metabolic processes, altered biosynthesis activity, and regulation of transcription factor activity. We found enrichment of proteins implicated in transcription factor activity regulation, which is pertinent in tendinopathy as gene expression drives matrix remodeling, inflammation, and fibrosis.^111^ Our platform also shows how EVs can play a role in altering metabolic processes in our disease model, where in tendinopathy, enhanced metabolic activity manifests due to the changing microenvironment.^112^ Tendinopathy also exhibits altered biosynthesis activity through enhanced cellular proliferation as well as altered ECM production.^112^, ^113^ All of these pathways are linked to hypoxia signaling, suggesting that hypoxic cues strongly shape EV cargo and cellular responses in our model. And in the context of tendinopathy, hypoxia plays a crucial role in the progression of disease by triggering inflammation, activating fibroblasts and altering collagen production.^114^

Collectively, these results underscore the utility of EVs as dynamic reporters and potential mediators of tendon remodeling upon the onset of injury or disease. The persistent enrichment of cytoskeletal, ECM, and immunomodulatory proteins in diseased EVs highlights their potential as candidate pathways that may be therapeutically targeted to interrupt or reverse fibrotic progression. Moreover, the differential temporal patterns observed in proteins DES, PEBP1, and ENO1 can highlight pathological stage‐specific molecular processes that could be leveraged to develop precision‐based interventions for early tendinopathy and fibrosis. Future studies should further explore if EVs having these proteomic signatures modulate recipient cell behavior and contribute to the perturbation of fibrosis in tendinopathy.

Our study has some limitations. We recognize that our models lack the critical soluble cues present in tendinopathy, as our model only depicts the structural changes in the matrix that occur with the onset of disease Future work will build upon our model to more closely resemble the complex biochemical features of healthy and diseased tendon tissue Furthermore, protein expression utilizing the capillary electrophoresis systems yielded low and inconsistent protein expression of our housekeeping gene, β‐actin. This may be due to an assay issue or the need for another housekeeping gene as β‐actin is widely used as a housekeeping gene in EVs, but its expression can vary due to stimuli including disease.^115^ The ECM is a dynamic regulator of tissue homeostasis and disease progression via bidirectional communication with resident cells, ultimately modulating cell behavior through cell–matrix interactions and remodeling processes. By sequestering soluble factors, including growth factors and matrix‐associated proteins, the ECM governs cellular proliferation, differentiation, migration, and apoptosis.^116^ Mechanical and topographical cues from the ECM further influence cell fate through mechanosignaling pathways. EVs contribute to this interplay by carrying matrix proteins and remodeling enzymes, directly impacting ECM structure and turnover, a previously established concept.^117^ ECM components and proteoglycans have been detected in circulating small EVs, with altered levels observed in disease, highlighting EVs as potential carriers of ECM‐derived signals.^118^ Moreover, EV–ECM interactions are reciprocal: the ECM regulates EV transport and uptake, while EV cargo modulates ECM composition and function.^119^ Although fibronectin, collagen I, and collagen III were not significantly enriched in our EV proteomics dataset, we identified several other ECM‐associated proteins, including versican, galectin‐1, and nidogen‐1, along with the remodeling enzyme MMP2, remodeling marker vimentin, and cell–matrix regulators THY1 and annexin A2. A potential limitation of the study is the interspecies differences between rats and humans in regard to ECM and EV profiles.^120^ ECM profiles differ between the species, with rats showing significant differences in COL1 gene expression compared to humans. Under inflammatory conditions, rat tendinous ECMs exhibit more pronounced decrease in COL1 relative to humans.^121^ In context to EVs, comparative proteomic analysis studies between human and animal EVs demonstrate a core set of conserved EV proteins (tetraspanins, ALIX, HSPs) in addition to species‐specific proteins.^122^ These findings highlight how many canonical markers are conserved across species, but candidate biomarker proteins or functional cargo require species‐specific validation. Therefore, future work will entail validation of our models with human tendon‐derived cells. Despite these limitations, our work highlights the temporal aspect of tendon disease, which is often overlooked in in vitro studies. This study captures the time‐dependent phenotypic shifts through our disease mimetic model, presenting a significant advancement in modeling tendinopathy. The temporal variations in EV cargo are reflective of the evolving cellular responses and pathological remodeling present in tendinopathy. Notably, the early enrichment of cytoskeleton and ECM‐associated proteins VIM, ANXA2, VCAN, and NID1 in diseased EVs by days 3 and 5 mirror the early activation of tendon fibroblasts and matrix deposition that occurs with tendinopathy onset. Furthermore, the sustained elevation of MMP2, ANXA1, and MFGE8 further suggest that diseased EVs contribute to persistent matrix remodeling, immune modulation, and fibrotic signaling, reinforcing the model's relevance to chronic tendon pathology. The proteomic profile at day 10 reveals a late‐stage phenotypic shift marked by enrichment of INHBA, VIM, ANXA1, DES, and ENO1, proteins associated with fibroblast activation, myofibroblast differentiation, stress responses, and metabolic reprogramming. The delayed emergence of these markers highlights the progressive nature of fibrotic remodeling and underscores the model's capacity to capture the temporal component of tendinopathy. This was especially evident in the divergent expression on day 10 of DES and PEBP1 proteins between healthy and diseased models. Our work reinforces that pathological tendon remodeling evolves over time and cannot be fully recapitulated in vitro through single‐timepoint or short‐term studies.

Our findings demonstrate that temporal analysis is crucial for understanding tendinopathy onset and progression. Our diseased models' ability to capture both early and late‐stage molecular signatures of tendinopathy cements it as a viable platform for studying dynamic disease processes. Through modeling healthy and diseased tendon microenvironments and characterizing changes in EV cargo, our study provides insights into the temporal mechanisms of tendon fibrosis and identifies EV‐mediated pathways as potential therapeutic targets. Moreover, our 3D model enables us to directly predict disease progression and evaluate targeted interventions for tendinopathy. Future investigations leveraging this system will aim to determine how temporal changes in EV cargo directly influence recipient cell behavior.

## 5 CONCLUSION

This study establishes a physiologically relevant 3D in vitro model that recapitulates key features of both healthy and fibrotic tendon microenvironments, elucidating their impact on tendon‐derived cell behavior and EV dynamics. Primary tendon‐derived cells exhibited distinct morphological, proliferative, and alignment profiles in response to healthy versus diseased mimetic platforms, consistent with phenotypes observed during tendon homeostasis and pathology. Notably, diseased‐mimetic scaffolds induced increased collagen III and fibronectin expression, as well as elevated proliferation, indicative of progressive matrix remodeling. Complementing these findings, proteomic profiling of EVs revealed temporally regulated cargo reflective of the underlying microenvironment. Diseased EVs were enriched in cytoskeletal, ECM‐remodeling, and inflammatory proteins, while healthy EVs maintained cargo associated with homeostatic function. These data suggest that EVs serve not only as sensitive indicators of cellular and matrix state but also as potential mediators of disease progression through paracrine signaling. Together, our results underscore the utility of this 3D tendon model for mechanistic studies of tendon pathology and repair. Furthermore, the identification of disease‐specific EV protein signatures provides a foundation for future investigations into the therapeutic potential of EVs in tendinopathy and other fibrotic disorders. Ongoing work will include in‐depth characterization of the miRNA cargo, coupled with functional studies to validate the contribution of EVs towards tendinopathic remodeling.

## AUTHOR CONTRIBUTIONS

Kariman Shama: conceptualization, formal analysis, investigation, methodology, project administration, validation, visualization, writing (original draft preparation, review & editing). Zachary F. Greenberg: formal analysis, investigation, data curation, visualization, writing (original draft preparation, review & editing). Evangeline Meyler: Investigation, data curation. Mei He: conceptualization, project administration, resources, supervision, writing (review & editing). Brittany Taylor: conceptualization, funding acquisition, project demonstration, resources, supervision, validation, writing (original draft preparation, review & editing).

## CONFLICT OF INTEREST STATEMENT

The authors declare no competing interests.

## Supporting information


**Figure S1:** Full membranes of western blot conducted on tendon derived cells isolated from monolayer, healthy and disease mimetic models. Molecular weight of fibronectin (~262 kDa), Collagen I (~138 kDa), Collagen III (~128 kDa), and β‐actin (~45 kDa).
**Figure S2:** Quantified graphs of (A) fibronectin, (B) collagen I, and (C) collagen III expression in tendon‐derived cells, normalized to β‐actin. Significance was defined as *p* < 0.05 (*), *p* < 0.01 (**), *p* < 0.001 (***), *p* < 0.0001 (****), ns = not significant.
**Figure S3:** Quantification of (A) fibronectin, (B) collagen I and (C) collagen III expression in EVs using the Jess capillary electrophoresis system. Protein expression was normalized to β‐actin. *p* < 0.05 (*), *p* < 0.01 (**), *p* < 0.001 (***), *p* < 0.0001 (****), ns = not significant.
**Figure S4:** (A) Nanoparticle tracking analysis (NTA) demonstrates particle concentration of extracellular vesicles isolated from monolayer control, healthy and diseased mimetic models. (B) MTS activity of tendon derived cels seeded on monolayer, healthy and diseased mimetic models. (C) Quantitative analysis of cells in our models was determined via nuclear staining. *p* < 0.05 (*), *p* < 0.01 (**), *p* < 0.001 (***), *p* < 0.0001 (****), ns = not significant.
**Figure S5:** Complete capillary electrophoresis panels showing the expression levels of collagen I (COL1A1), collagen III (COL3A1), fibronectin, and β‐actin proteins on (A) day 3, (B) day 5, (C) day 7 and (D) day 10.
**Figure S6:** Line plots showing the time series analysis of non‐statistically significant proteins tendon‐derived EVs carry.
**Figure S7:** Day 7 differential analysis between healthy and disease cells.
**Figure S8:** (A) Transmission electron microscopy (TEM) was conducted on isolated particles to demonstrate spherical morphology conducive to EVs taken at 150 kX. (B) Nanoparticle tracking analysis demonstrates size distribution of isolated EVs among the three groups. (C) Expression of TSG101, CD63 and CD81 in isolated extracellular vesicles.

**Table S1:** Summary of enrichment analysis from proteomics dataset, including source database, term name, term identifier, adjusted *p*‐value, the number of proteins queried (query size), the number of proteins overlapping with each term (intersection size), the number of proteins overlapping with each term (intersection size) and list of intersecting proteins identified in our dataset.

## Data Availability

Computer code used in this manuscript is available at https://github.com/zfg2013/Tendinopathy. All code and proteomic data used in this manuscript are included in the repository. To ensure reproducibility and facilitate easy use, we provide a README with version information for each tool plus any parameter settings used in the data processing and analysis. The online version contains supplementary material. The supplemental material includes full, uncropped Western blot membranes, additional time‐series analyses from proteomic datasets presented as graphical plots, general extracellular vesicle characterization data, and supplementary cell viability assay results. Table S1 provides analyzed functional pathways associated with DES, ENO1, GDI2, and PEBP1 using Gene Ontology.
