# Supplementary material for: Extracellular matrix microarchitecture modulates cellular behavior and extracellular vesicle phenotypes in biomimetic tendon models
Source: Bioeng Transl Med. 2026 Apr 24;11(3):e70134. doi: 10.1002/btm2.70134 (PMC13247418; doi:10.1002/btm2.70134)
Supplement: Supplementary file 1 — Figure S1: Full membranes of western blot conducted on tendon derived cells isolated from monolayer, healthy and disease mimetic models. Molecular weight of fibronectin (~262 kDa), Collagen I (~138 kDa), Collagen III (~128 kDa), and β‐actin (~45 kDa). Figure S2: Quantified graphs of (A) fibronectin, (B) collagen I, and (C) collagen III expression in tendon‐derived cells, normalized to β‐actin. Significance was defined as p < 0.05 (*), p < 0.01 (**), p < 0.001 (***), p < 0.0001 (****), ns = not significant. Figure S3: Quantification of (A) fibronectin, (B) collagen I and (C) collagen III expression in EVs using the Jess capillary electrophoresis system. Protein expression was normalized to β‐actin. p < 0.05 (*), p < 0.01 (**), p < 0.001 (***), p < 0.0001 (****), ns = not significant. Figure S4: (A) Nanoparticle tracking analysis (NTA) demonstrates particle concentration of extracellular vesicles isolated from monolayer control, healthy and diseased mimetic models. (B) MTS activity of tendon derived cels seeded on monolayer, healthy and diseased mimetic models. (C) Quantitative analysis of cells in our models was determined via nuclear staining. p < 0.05 (*), p < 0.01 (**), p < 0.001 (***), p < 0.0001 (****), ns = not significant. Figure S5: Complete capillary electrophoresis panels showing the expression levels of collagen I (COL1A1), collagen III (COL3A1), fibronectin, and β‐actin proteins on (A) day 3, (B) day 5, (C) day 7 and (D) day 10. Figure S6: Line plots showing the time series analysis of non‐statistically significant proteins tendon‐derived EVs carry. Figure S7: Day 7 differential analysis between healthy and disease cells. Figure S8: (A) Transmission electron microscopy (TEM) was conducted on isolated particles to demonstrate spherical morphology conducive to EVs taken at 150 kX. (B) Nanoparticle tracking analysis demonstrates size distribution of isolated EVs among the three groups. (C) Expression of TSG101, CD63 and CD81 in isolated extracellular vesicle [file BTM2-11-e70134-s002.docx]

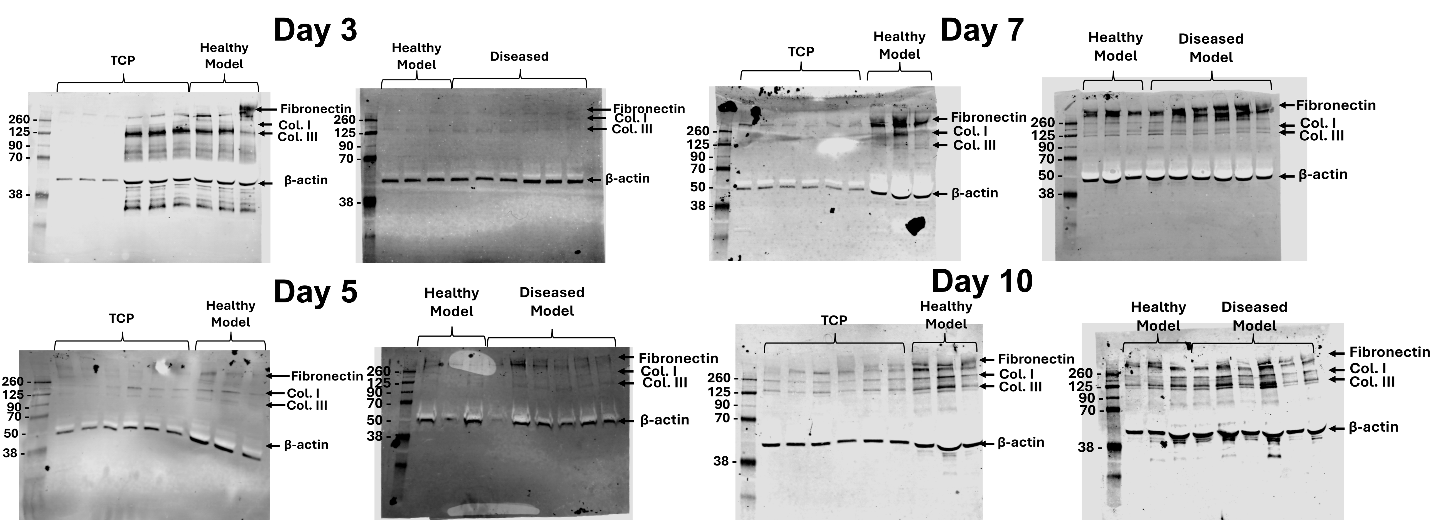
Supplemental Materials

Supplementary Figure 1: Full membranes of western blot conducted on tendon derived cells isolated from monolayer, healthy and disease mimetic models. Molecular weight of fibronectin (~262 kDa), Collagen I (~138 kDa), Collagen III (~ 128 kDa), and β-actin (~45 kDa).


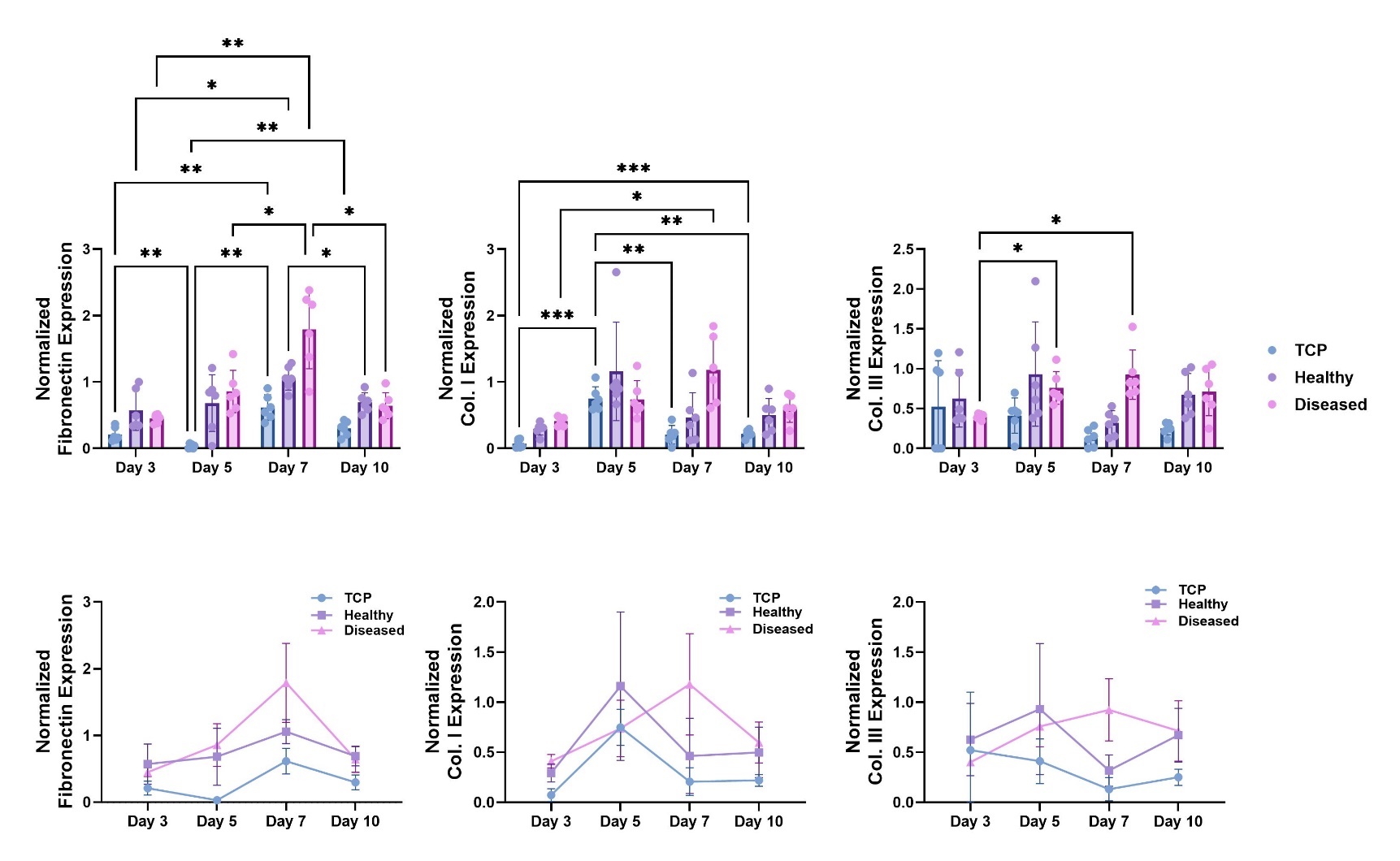


Supplementary Figure 2: Quantified graphs of (A) fibronectin, (B) collagen I, and (C) collagen III expression in tendon-derived cells, normalized to β-actin. Significance was defined as p < 0.05 (*),  p < 0.01 (**), p < 0.001 (***), p < 0.0001 (****), ns= not significant.

**A.**

**B.**

**C.**


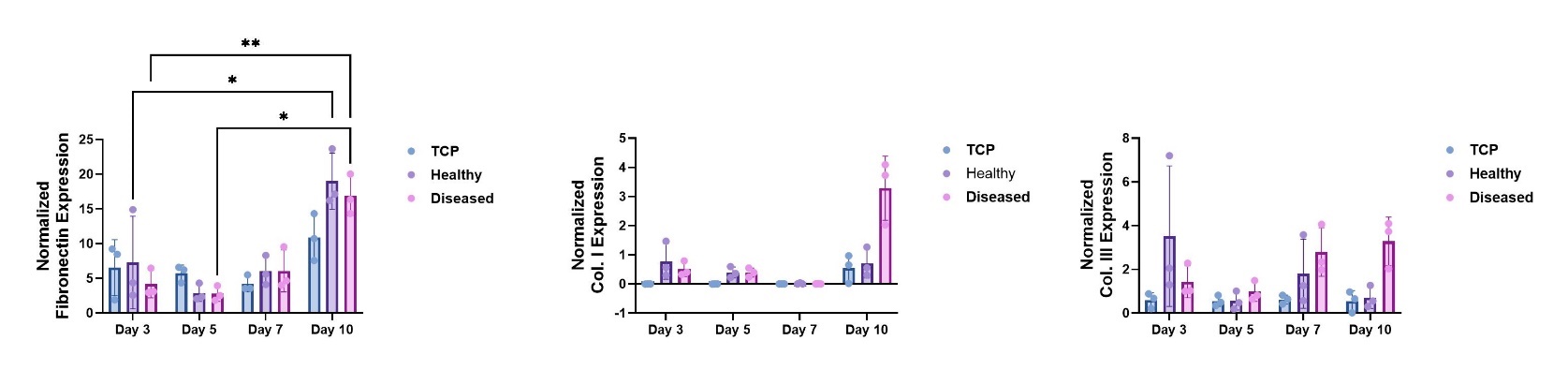


Supplementary Figure 3: Quantification of (A) fibronectin, (B) collagen I and (C) collagen III expression in EVs using the Jess capillary electrophoresis system. Protein expression was normalized to β-actin. p < 0.05 (*),  p < 0.01 (**), p < 0.001 (***), p < 0.0001 (****), ns= not significant.

**C.**

**B.**

**A.**


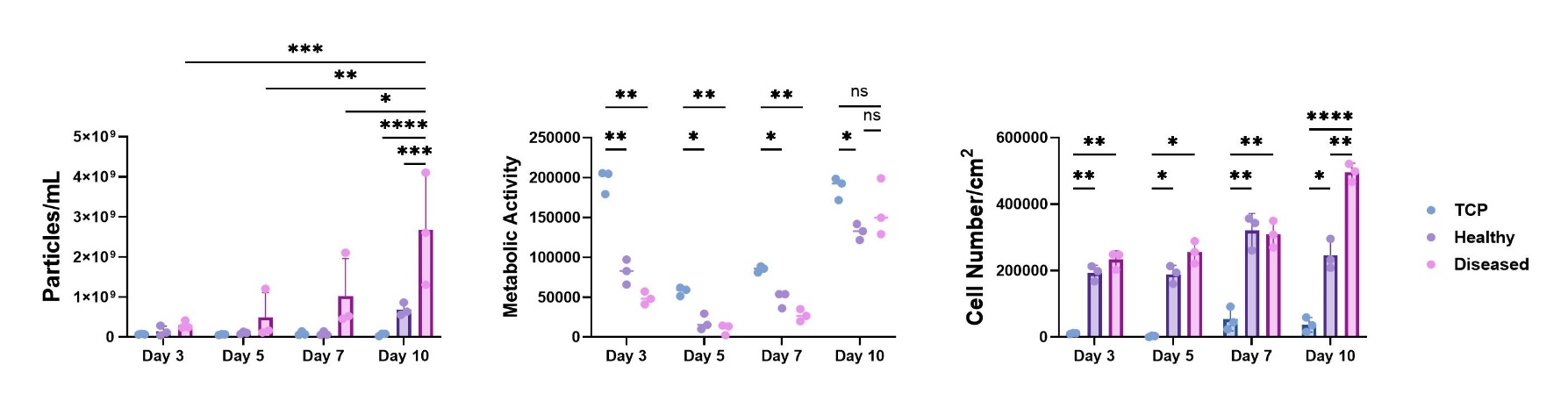


Supplementary Figure 4: (A) Nanoparticle tracking analysis (NTA) demonstrates particle concentration of extracellular vesicles isolated from monolayer control, healthy and diseased mimetic models. (B) MTS activity of tendon derived cels seeded on monolayer, healthy and diseased mimetic models. (C) Quantitative analysis of cells in our models was determined via nuclear staining. p < 0.05 (*),  p < 0.01 (**), p < 0.001 (***), p < 0.0001 (****), ns= not significant.

**A.**

**B.**

**C.**

Figure 3: Full Jess panels for each marker at each timepoint utilizing the Jess capillary electrophoresis system.


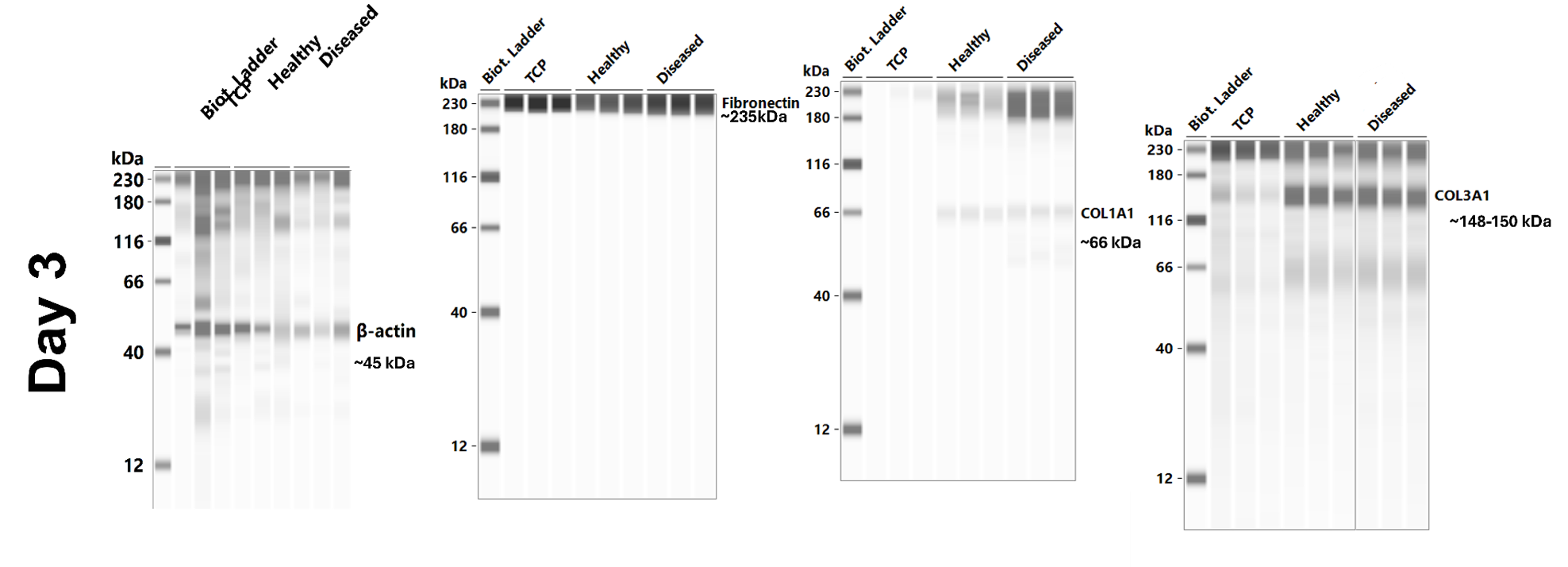

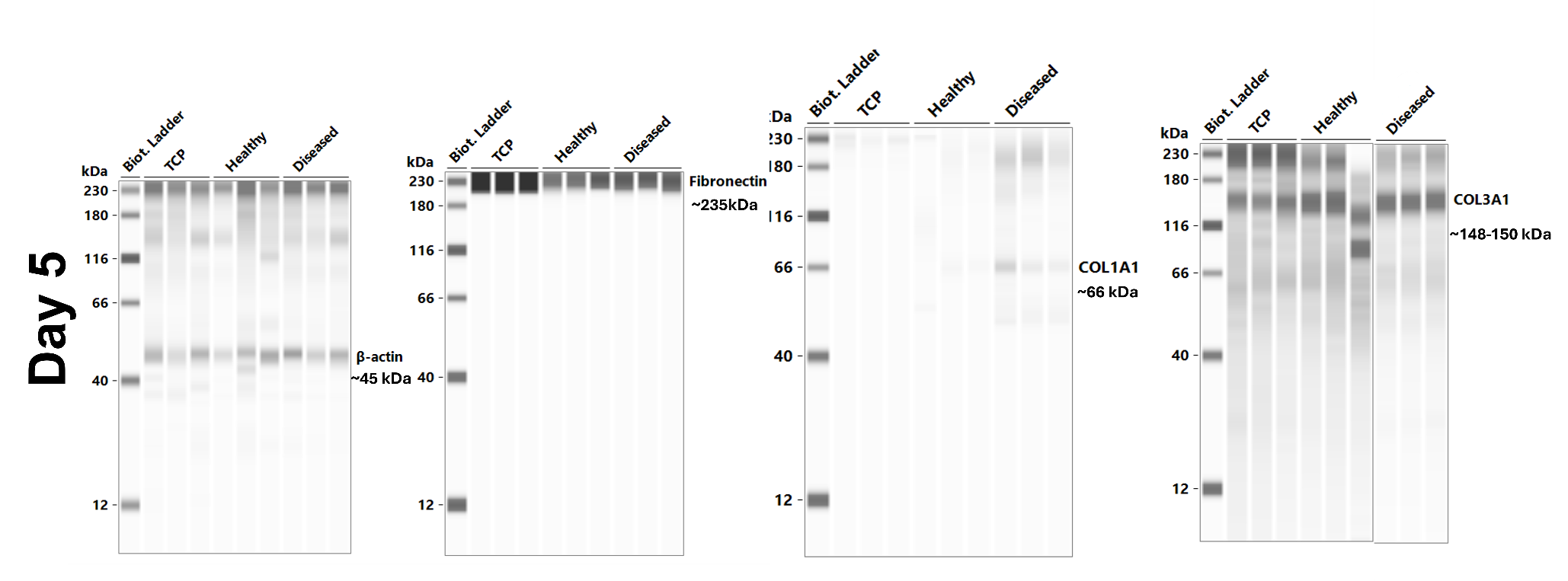


**A.**

**B.**


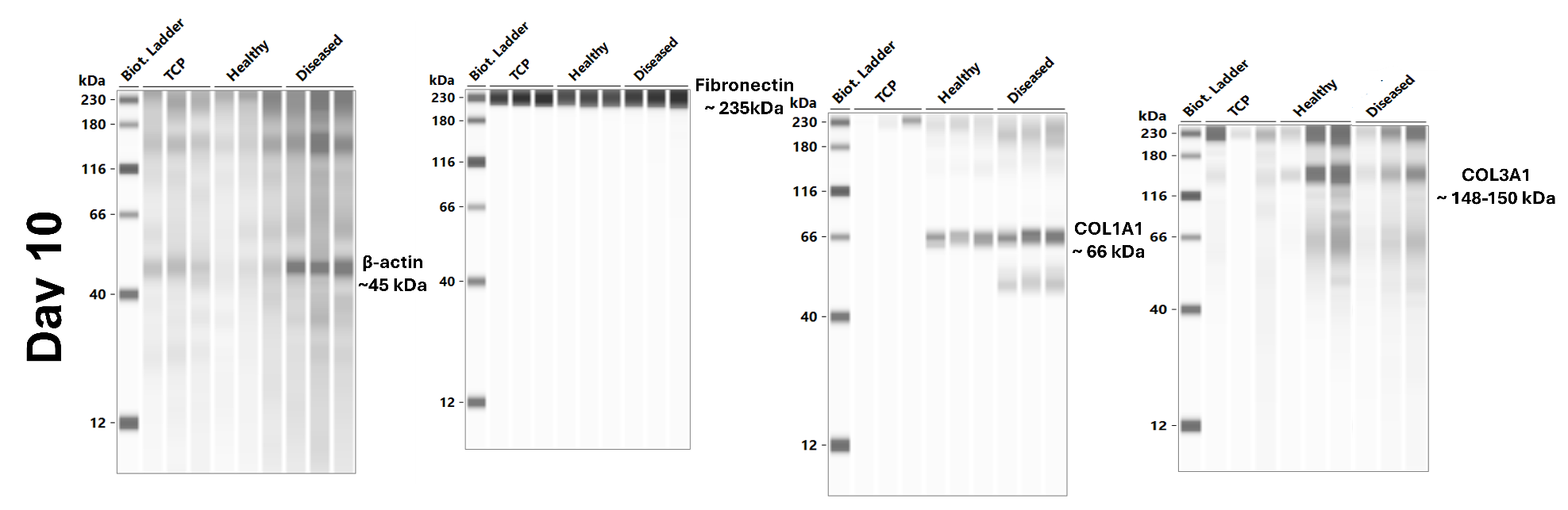

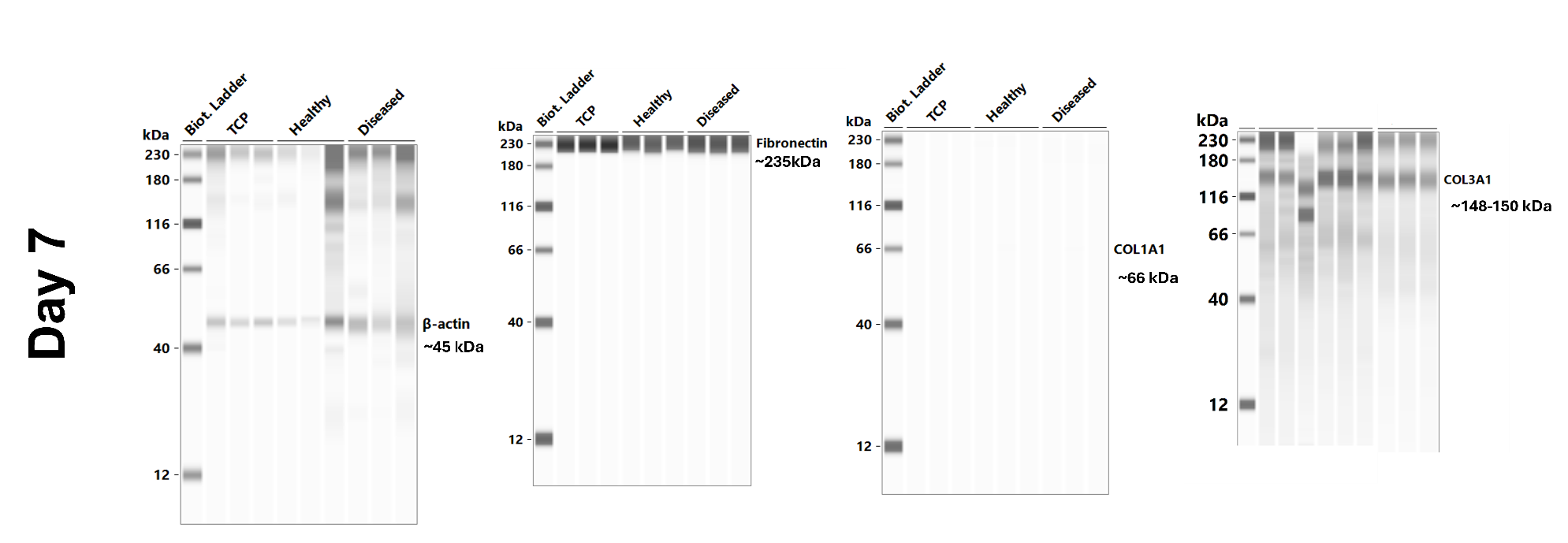


**D.**

**C.**

Supplementary Figure 5: Complete capillary electrophoresis panels showing the expression levels of collagen I (COL1A1), collagen III (COL3A1), fibronectin, and β-actin proteins on (A) day 3, (B) day 5, (C) day 7 and (D) day 10.


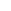

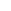

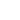


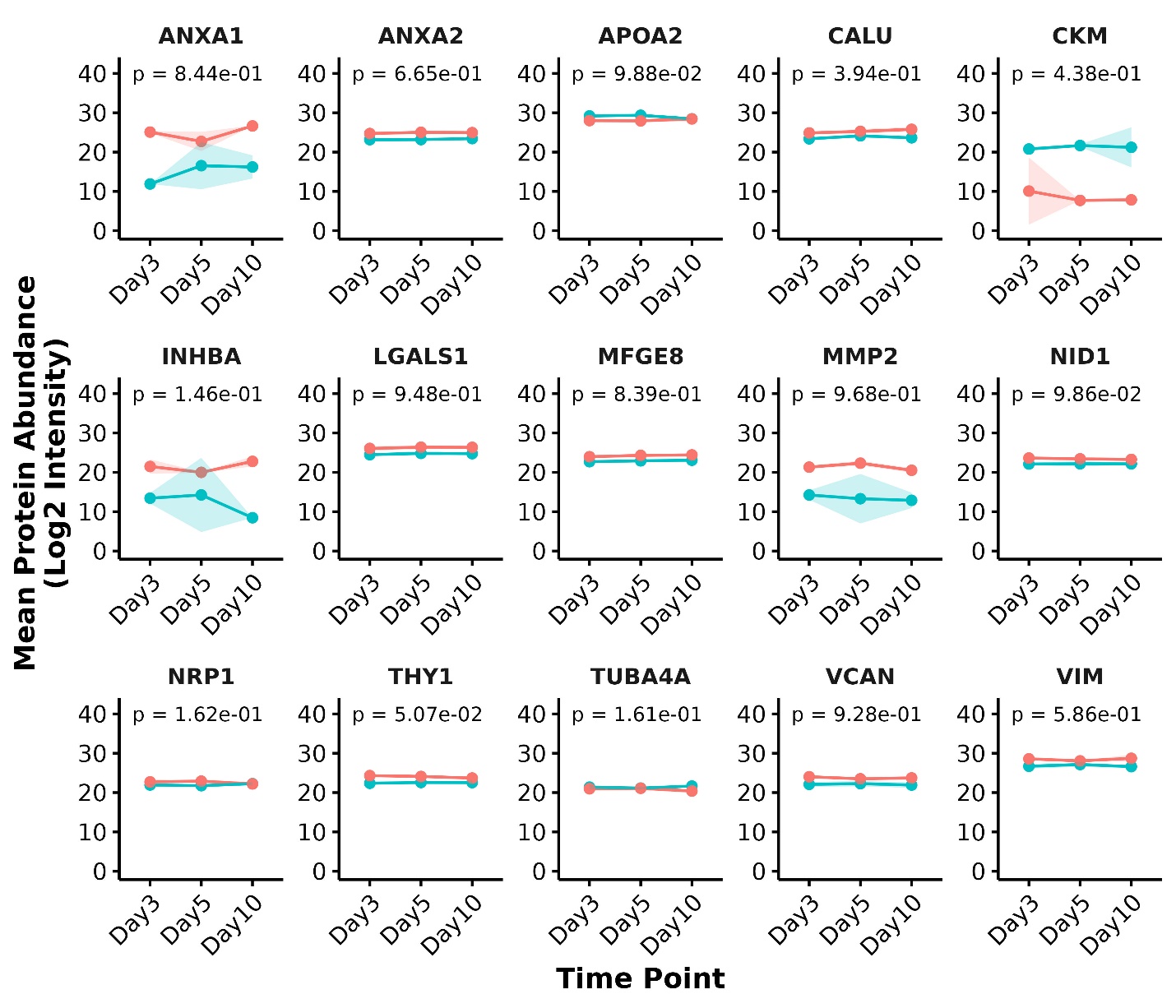


Supplementary Figure 6. Line plots showing the time series analysis of non-statistically significant proteins tendon-derived EVs carry.


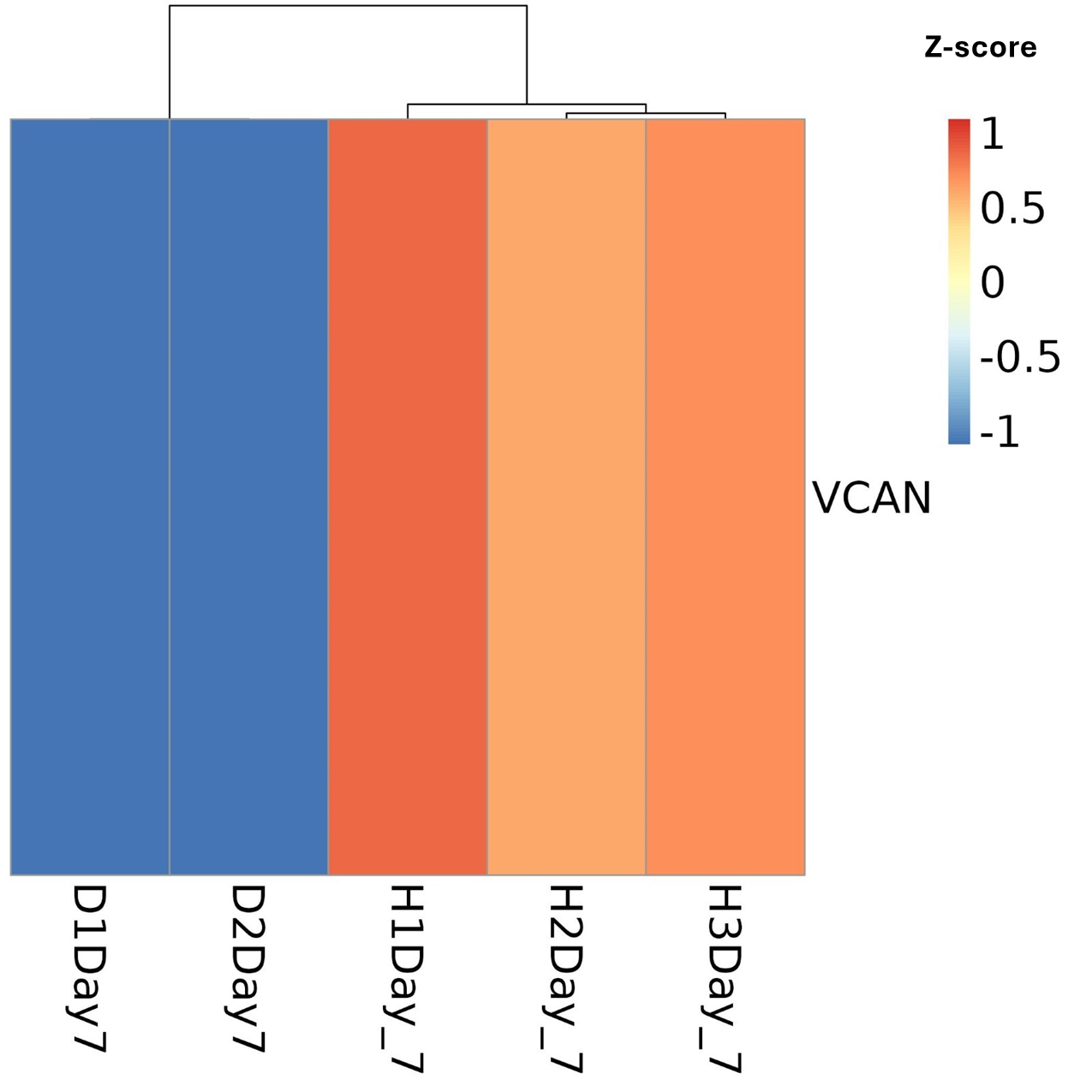


Supplementary Figure 7. Day 7 differential analysis between healthy and disease cells.


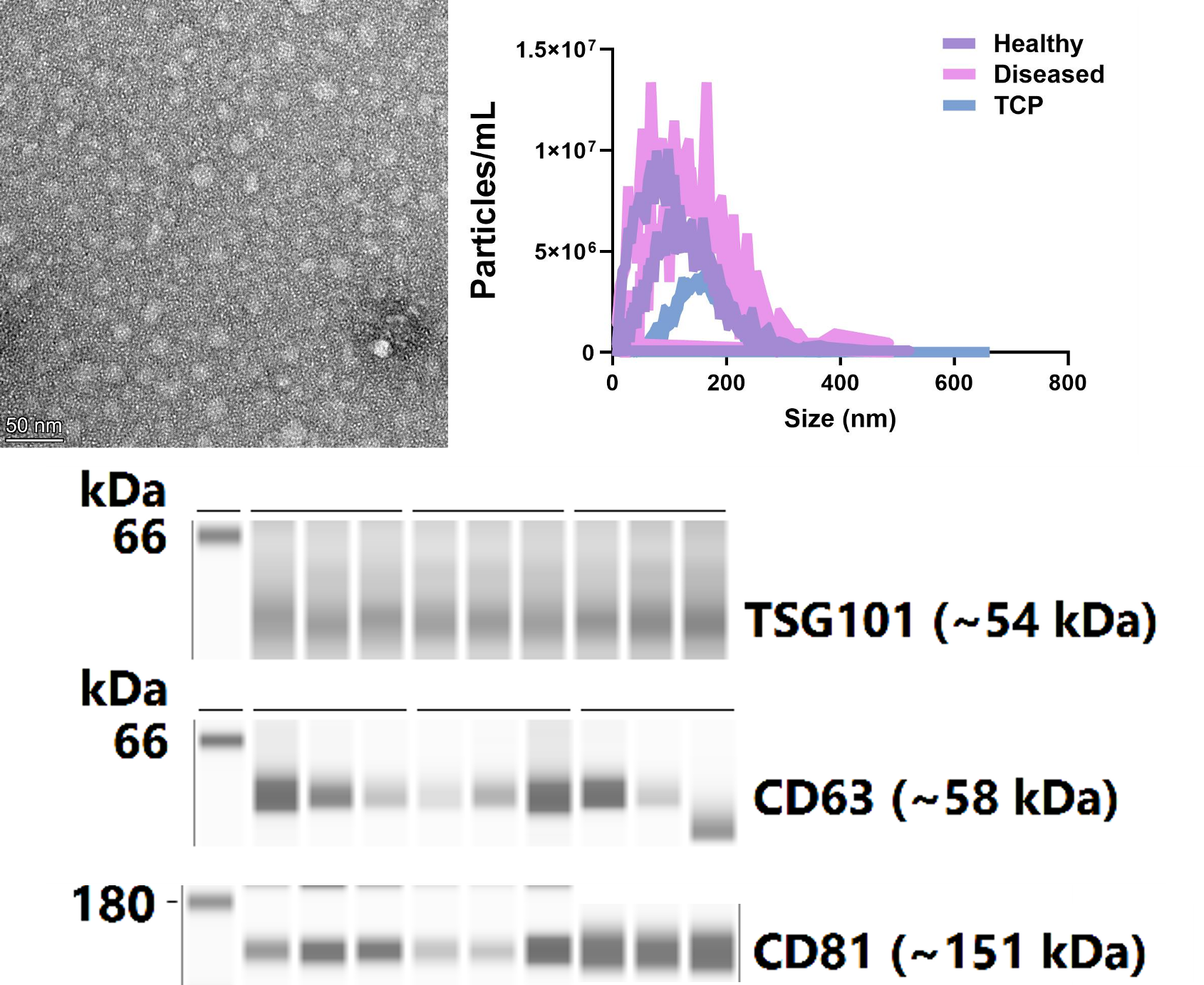


**TCP**

**Healthy**

**Diseased**

**A.**

**B.**

**C.**

Supplementary Figure 8: (A) Transmission electron microscopy (TEM) was conducted on isolated particles to demonstrate spherical morphology conducive to EVs taken at 150kX. (B) Nanoparticle tracking analysis demonstrates size distribution of isolated EVs amongst the three groups. (C) Expression of TSG101, CD63 and CD81 in isolated extracellular vesicles.
